# Evaluation of analgesic and anti-inflammatory activities of the root extract of *Grewia schweinfurthii* Burret and its major chemical constituents

**DOI:** 10.1371/journal.pone.0353400

**Published:** 2026-07-22

**Authors:** Abdi Leta Gemechu, Mirutse Giday, Solomon Tadesse, Ariaya Hymete

**Affiliations:** 1 Ambo University, College of Medicine and Health Science, Department of Pharmacy, Ambo, Ethiopia; 2 Addis Ababa University, College of Health Sciences, Department of Pharmaceutical Chemistry and Pharmacognosy, Addis Ababa, Ethiopia; 3 Addis Ababa University, Aklilu Lemma Institute of Pathobiology, Addis Ababa, Ethiopia; 4 Department of Biomedical and Pharmaceutical Sciences, L. S. Skaggs College of Pharmacy, Kasiska Division of Health Sciences, Idaho State University, Pocatello, Idaho, United States of America; The kids Research Institute Australia, AUSTRALIA

## Abstract

Despite the widespread use of conventional analgesic and anti-inflammatory drugs, their clinical utility is often limited by adverse effects, necessitating the search for safer alternatives from medicinal plants. This study investigated the analgesic and anti-inflammatory activities of the 80% methanolic root extract of *Grewia schweinfurthii* Burret and an isolated bioactive compound using established rodent models. Air-dried roots were extracted by maceration with 80% methanol, and acute oral toxicity was evaluated following OECD guidelines. Analgesic activity was assessed using the acetic acid–induced writhing and hot plate models in mice, while anti-inflammatory activity was evaluated using carrageenan-induced paw edema in rats. A bioactive compound, 4 (2ʺ-(4′-isopropylphenyl) propan-2ʺ-yl)-2,3-dihydrofuran, was isolated via column chromatography and tested for anti-inflammatory activity. In the writhing test, the extract produced significant (p < 0.001) dose-dependent inhibition of abdominal constrictions, with 13.90%, 56.81%, and 75.48% inhibition at 100, 200, and 400 mg/kg, respectively, compared to 80.77% inhibition by aspirin (150 mg/kg). In the hot plate model, the 400 mg/kg dose significantly prolonged latency time from a baseline of 5.67 ± 0.33 s to 9.17 ± 1.01 s at 120 min (p < 0.05), indicating central analgesic activity. In the carrageenan-induced paw edema model, the extract demonstrated marked anti-inflammatory effects, with 93% inhibition at 400 mg/kg at the 5th hour, comparable to indomethacin (95% inhibition). The isolated compound exhibited significant dose-dependent anti-inflammatory activity, achieving 75% inhibition at 40 mg/kg at 5 hours (p < 0.001). Overall, the findings demonstrate that *G. schweinfurthii* root extract and its isolated compound possess significant peripheral and central analgesic as well as potent anti-inflammatory activities, supporting the plant’s traditional use; however, further mechanistic, toxicological, and pharmacokinetic studies are required before clinical relevance can be established.

## Introduction

### Background

Pain is an unpleasant sensory and emotional experience related to or resembling an actual or potential tissue injury [1]. Although unpleasant, pain serves an essential protective function by acting as a warning signal of injury or disease [2]. Nearly 100 million people in the United States suffer from chronic pain, with estimated annual economic costs ranging between $560 and $635 billion due to healthcare expenditures and lost productivity [3]. Globally, pain represents a major public health burden, with approximately 20% of adults reporting persistent pain and 10% developing new chronic pain annually [4].

Inflammation is a complex biological response of the immune system to harmful stimuli such as pathogens, damaged cells, or irritants, aiming to eliminate the initial cause of injury and initiate tissue repair [5]. At the tissue level, it is characterized by vascular dilation, increased vascular permeability, leukocyte infiltration, and the release of inflammatory mediators. While inflammation is protective in the short term, uncontrolled or excessive inflammatory responses can lead to tissue damage and contribute to the progression of numerous acute and chronic diseases, including rheumatoid arthritis, atherosclerosis, psoriasis, inflammatory bowel disease, retinitis, and multiple sclerosis [6].

Acute inflammation is a rapid and early response to harmful stimuli, marked by plasma exudation and predominantly neutrophilic leukocyte infiltration. While acute inflammation is a necessary and beneficial component of host defense, a dysregulated or excessively prolonged acute inflammatory response can lead to collateral tissue injury and contribute to worse disease outcomes [3]. Chronic inflammation, in contrast, is a prolonged process lasting weeks to months, characterized by the simultaneous occurrence of persistent inflammation, tissue destruction, and repair attempts [4].

Pain and inflammation frequently coexist in many pathological conditions. Opioids and nonsteroidal anti-inflammatory drugs (NSAIDs) remain the cornerstone of therapy; however, their long-term use is limited by significant adverse effects such as gastrointestinal injury, renal toxicity, cardiovascular risk, and opioid dependence or respiratory depression [7–10].

Medicinal plants have long been used to manage pain and inflammatory disorders, and increasing scientific evidence supports their pharmacological potential [11]. Phytochemicals such as flavonoids, tannins, saponins, alkaloids, and terpenoids are known to exert analgesic and anti-inflammatory effects through mechanisms including inhibition of cyclooxygenase (COX) and lipoxygenase pathways, suppression of pro-inflammatory cytokines, antioxidant activity, and modulation of nociceptive signaling pathways. Several plant species have demonstrated promising anti-inflammatory and analgesic activities; however, rather than listing multiple species, accumulating evidence indicates that plant-derived bioactive compounds represent an important reservoir for safer therapeutic agents [6,12–15].

*Grewia schweinfurthii* Burret (Malvaceae) is a shrub widely distributed in Ethiopia and other parts of Africa. Its fruits are commonly consumed as food in Ethiopia, and various parts of the plant are traditionally used to manage conditions associated with pain and inflammation, including toothache, headache, joint pain, wounds, and fever [16–24]. Although several species of the genus *Grewia* have been investigated pharmacologically [24,25], there is limited experimental evidence validating the analgesic and anti-inflammatory activities of *G. schweinfurthii*, particularly regarding its root extract and its bioactive constituents. Moreover, the specific compounds responsible for these traditional effects remain largely unidentified. Furthermore, no study to date has simultaneously evaluated both the crude extract and a purified bioactive compound from this species using validated peripheral, central, and inflammatory pain models. The present study therefore addresses this gap by providing the first systematic pharmacological characterization of G. schweinfurthii root and its major isolated compound, AL-03, using complementary in vivo models — representing the first dual extract-compound pharmacological evaluation of this species.

Therefore, the present study aimed to (i) evaluate the analgesic activity of the 80% methanolic root extract of *G. schweinfurthii* using both peripheral and central pain models, (ii) assess its anti-inflammatory activity using the carrageenan-induced paw edema model, and (iii) isolate and characterize the major bioactive compound responsible for the observed pharmacological effects.

## Methods

### Materials

#### Plant material.

The roots of *G. schweinfurthii* Burret were collected in June 2022 from Awash National Park, located approximately 231 km east of Addis Ababa. The plant material was taxonomically identified by Mr. Melaku Wondafrash at the National Herbarium, College of Natural and Computational Sciences, Addis Ababa University. A voucher specimen (Voucher No.: AL001/2022) was prepared and deposited at the National Herbarium, Addis Ababa University, Ethiopia, for future reference.

#### Chemicals, reagents and drugs.

Methanol, n-hexane, ethyl acetate, and chloroform (all obtained from Sigma-Aldrich Co., MO, USA) were utilized for extraction and chromatography. For analytical TLC, Pre-coated silica gel 60 F254 plates (aluminum-backed, 200 µm thickness, Merck KGaA, Darmstadt, Germany) were used. were employed, while silica gel F254 was used for preparative TLC. Carrageenan (Sigma Chemicals Co., St. Louis, USA), normal saline (H. R. Leuven, Belgium), distilled water, glacial acetic acid (Sigma-Aldrich Laborchemikalien, Germany), indomethacin (Cadila Pharmaceuticals, Ethiopia), as well as aspirin and morphine (Ethiopian Pharmaceutical Manufacturing Factory, Ethiopia) were used in the experiment.

#### Instrument.

Digital plethysmometer, hot plate and electronic balance (Orchid Scientific, India), rotary evaporator (Heidolph, Germany), UV spectrophotometer, nuclear magnetic resonance spectrometer (Bruker Avance DMx400 FT-NMR spectrometer using TMS as internal standard), drying oven and fume hood were used in the experiment.

#### Experimental animals.

Healthy Swiss albino mice (6–8 weeks old) and Wistar albino rats (6–8 weeks old) of both sexes were used in the study. The animals were kept under standard laboratory conditions with a 12-hour light-dark cycle and provided free access to food and water. To reduce stress, the mice were allowed a one-week acclimatization period to the laboratory environment prior to the start of the experiments. All procedures involving animal were carried out in compliance with internationally recognized guidelines for the ethical use of laboratory animals and were approved by Ethical Review Board of School of Pharmacy of the Addis Ababa University. (Approved code: ERB/SOP/538/15/2023) [26].

#### Animal care, ethical approval, and humane endpoints.

Animals were monitored regularly for general health status, behavior, and signs of pain or distress throughout the experimental period. Observations were conducted at least twice daily and more frequently during the acute oral toxicity study and immediately following experimental interventions. Humane endpoints were predefined prior to study initiation to minimize animal suffering. Animals were humanely euthanized if they exhibited severe or persistent signs of distress or toxicity, including marked body weight loss (>20%), lethargy, inability to access food or water, severe diarrhea, tremors, paralysis, abnormal posture, or any other indication of poor prognosis.

All experimental procedures were designed to minimize pain and distress. Where applicable, appropriate handling techniques were employed to reduce stress during drug administration and experimental measurements. No surgical procedures were performed, and therefore no additional anesthetic or analgesic agents were required during the experimental procedures.

At the end of the experimental procedures, animals were humanely euthanized using an overdose of ketamine (100 mg/kg) and xylazine (10 mg/kg) administered intraperitoneally to ensure rapid loss of consciousness and minimal suffering, in accordance with internationally accepted animal welfare guidelines. Death was subsequently confirmed by a secondary physical method (e.g., cervical dislocation or bilateral thoracotomy) to ensure complete euthanasia.

All possible efforts were made to minimize pain and distress, and the number of animals used was kept to the minimum required to achieve statistical validity, in accordance with the principles of Replacement, Reduction, and Refinement (3Rs).

This study is reported in accordance with the ARRIVE (Animal Research: Reporting of *In Vivo* Experiments) guidelines, and the completed ARRIVE checklist is provided as Supporting Information.

### Ethics approval

All experimental procedures involving animals were reviewed and approved by the Ethical Review Board of the School of Pharmacy, Addis Ababa University, Ethiopia (Approval No.: ERB/SOP/538/15/2023).

### Methods

#### Preparation of plant material.

The collected roots of *G. schweinfurthii* were thoroughly washed with distilled water, air-dried at room temperature, and ground into a coarse powder using a mechanical grinder. The powdered plant material was extracted by maceration with 80% methanol for 72 h at room temperature with occasional shaking. After filtration through Whatman No. 1 filter paper, the marc was re-macerated twice with fresh solvent under the same conditions to maximize extraction efficiency. The combined filtrates were concentrated under reduced pressure using a rotary evaporator and subsequently lyophilized to obtain the crude extract. The extraction yield was 12.6% (w/w) relative to the dried plant material. The dried extract was stored in airtight amber-colored glass containers at 4 °C and protected from light and moisture until further experimental use.

#### Preliminary phytochemical screening.

The 80% methanol root extract of *G. schweinfurthii* was subjected to phytochemical screening to determine the presence or absence of secondary metabolites such as anthraquinones, cardiac glycosides, saponins, steroids, tannins, terpenoids and flavonoids using standard procedures [27].

#### Isolation of the major compound.

Column chromatography was performed using silica gel as the stationary phase. The column was initially packed with a slurry of silica gel prepared in chloroform (CHCl_3_). The sample was adsorbed onto silica gel by mixing the crude root extract (2 g) with silica gel (2 g) in methanol (MeOH), followed by drying under reduced pressure. The dried adsorbed sample was carefully loaded onto the top of the column and eluted using gradient mixtures of CHCl_3_ and MeOH. This solvent system was selected to facilitate polarity-based separation, where chloroform preferentially elutes non-polar constituents while increasing proportions of methanol enable the elution of moderately polar to polar compounds. The process resulted in 120 fractions (each 10 ml). The fractions were collected as follows: 1–10 Frs, CHCl_3_ (100%); 11–20 Frs, CHCl_3_-MeOH (95:5); 21–30 Frs, CHCl_3_-MeOH (90:10); 31–40 Frs, CHCl_3_-MeOH (85:15); 41–50 Frs, CHCl_3_-MeOH (80:20); 51–60 Frs, CHCl_3_-MeOH (75:25); 61–70 Frs, CHCl_3_- MeOH (70:30); 71–80 Frs, CHCl_3_-MeOH (65:35); 81–90 Frs, CHCl_3_-MeOH (60:40); 91–100 Frs, CHCl_3_-MeOH (55:45); 101–110 Frs, CHCl_3_-MeOH (50:50); 110–120 Frs, MeOH (100%). Contents of all fractions were monitored using TLC under a UV light at 254 and 366 nm. Fractions 21–30, which displayed a single spot-on TLC (CHCl_3:_ MeOH (6:1), and CHCl_3_: EA (2:1)) were combined to yield a white amorphous compound, coded AL-03. AL-03 was selected for further pharmacological evaluation based on three criteria: (i) it was the principal compound isolated from the active chromatographic fractions (fractions 21–30); (ii) TLC analysis in two independent solvent systems (CHCl_3_:MeOH 6:1 and CHCl_3_:EtOAc 2:1) consistently yielded a single spot, confirming chromatographic purity; and (iii) it represented the highest-yielding isolate from column chromatography, suggesting it is a major constituent of the root extract. Structural elucidation of AL-03 was subsequently performed using ^1^H and ^13^C NMR spectroscopy, and its identity is described in the Results section. **The detailed figures are presented in Supporting Information (S4–S6 Figs in** **S1 File****).**

#### Spectroscopic techniques.

Nuclear magnetic resonance (NMR) spectra were recorded on a Bruker Avance DMX-400 FT-NMR spectrometer (Bruker BioSpin, Germany) operating at 400 MHz for ¹H NMR and 100 MHz for ^13^C NMR. One-dimensional (^1^H and ^13^C) and two-dimensional HSQC experiments were performed to assist structural elucidation of the isolated compound. Samples were dissolved in deuterated chloroform (CDCl_3_) at an approximate concentration of 10 mg/mL. Chemical shifts (δ) were referenced to tetramethylsilane (TMS) as an internal standard and are reported in parts per million (ppm), while coupling constants (J) are expressed in Hertz (Hz). Spectra were acquired at room temperature (approximately 25 °C) without solvent signal suppression.

For ^1^H NMR analysis, the spectral region from 0–12 ppm was scanned, whereas ^13^C NMR spectra were recorded over the range of 0–205 ppm. Signal multiplicities in the ^1^H NMR spectra are described as s (singlet), d (doublet), t (triplet), q (quartet), dd (doublet of doublets), and m (multiplet). The structure of the isolated compound was elucidated based on the combined interpretation of ^1^H NMR, ^13^C NMR, and HSQC spectra, and the full spectra are provided in the Supporting Information.

#### Acute oral toxicity test.

Acute oral toxicity testing was conducted using healthy Swiss albino mice (6–8 weeks old, 25–30 g), according to OECD Guideline 423 [26]. On the first day, a single dose of 2000 mg/kg was given to a fasting mouse, after which four additional mice were treated in sequence depending on the outcome observed in the initial animal. The mice were carefully monitored for toxic signs, including weight loss, diarrhea, tremors, lethargy, and paralysis, during the first four hours and throughout the 24-hour period, followed by daily observation for 14 days to detect any signs of toxicity or death. Based on the absence of mortality at 2000 mg/kg, three dose levels were selected for pharmacological evaluation: 100, 200, and 400 mg/kg (corresponding to 1/20th, 1/10th, and 1/5th of the limit dose, respectively).

#### Animal grouping and dosing.

Wistar albino rats weighing 200–300 g (for assessing anti-inflammatory activity) and Swiss albino mice of either sex weighing 25–35 g (for evaluating analgesic activity) was randomly assigned into five groups, each consisting of six animals. Group I served as the negative control and received distilled water, while Group II acted as the positive control, receiving standard drugs: aspirin (150 mg/kg) for the acetic acid-induced writhing test, morphine (10 mg/kg) for the hot plate test, and indomethacin (10 mg/kg) for carrageenan-induced paw edema. The remaining three groups (test groups) were administered different doses (100, 200, and 400 mg/kg) of the methanol extract orally. All extracts and standard drugs were administered orally (p.o.), except morphine, which was administered intraperitoneally (i.p.). Additionally, the pure compound at doses of 10, 20, and 40 mg/kg was tested for anti-inflammatory activity using the carrageenan-induced paw edema model. Animals were allocated to groups by random number assignment. The group size of n = 6 per group was determined based on the sample sizes used in comparable published studies employing the same models; formal a priori power analysis was not performed and is acknowledged as a limitation. Animals of both sexes were included; sex was distributed as evenly as possible across groups, but no formal sex-stratification or sex-based subgroup analysis was performed. Sex-related differences in nociceptive and inflammatory responses are recognized in the literature and represent a limitation that future studies should address with appropriately powered, sex-stratified designs. All behavioral assessments (writhing counts, hot plate latency) were performed by an investigator blinded to group assignment.

#### Evaluation of analgesic activity of the extract.

**Acetic acid-induced writhing test:** The peripheral analgesic activity of the extract was evaluated using the method described by Ayanaw *et al*, [4]. Mice were randomly assigned into five groups, each consisting of six animals, with free access to water and subjected to overnight fasting. One hour prior to intraperitoneal administration of acetic acid (0.6% v/v, 10 ml/kg), the mice were treated orally with the crude extract at doses of 100, 200, or 400 mg/kg, while the negative control group received distilled water and the positive control group received aspirin (150 mg/kg). Five minutes after the acetic acid injection, the extent of writhing (specifically, the contraction of abdominal muscles and extension of hind limbs) was observed and recorded for 20 minutes to assess the analgesic effect of the extract. The analgesic potential of the extract was demonstrated by a reduction in the number of writhes compared to the control group, and expressed as a percentage inhibition of writhing as follows.


$$\begin{array}{c} \%\text{inhibition}=\frac{\text{mean writhing count }(\text{control group}-\text{treated group})}{\text{mean writhing count control group }}\times \text{1}\text{0}\text{0} \end{array}$$

**Hot plate method:** The central analgesic activity of the 80% methanolic root extract of *G. schweinfurthii* was evaluated using the hot plate test. In this assay, mice were individually placed in an open-ended cylindrical chamber positioned on a metal plate maintained at 55 ± 0.5 °C. Nociceptive responses were assessed by measuring the latency to paw licking or jumping, which are considered supraspinally mediated responses.

Baseline latency was recorded for each mouse prior to treatment, and animals with baseline latencies greater than 15 s were excluded from the experiment. A cut-off time of 15 s was applied to prevent tissue damage. One hour after oral administration of the extract (100, 200, and 400 mg/kg), the standard drug, or distilled water (control), the response latency was recorded. Reaction times were measured at 0, 30-, 60-, 90-, and 120-min following treatment. Analgesic activity was evaluated based on the prolongation of response latency compared with the control group [2].

#### Anti-inflammatory activity.

**Carrageenan-induced paw edema:** The *in vivo* anti-inflammatory activity of the extract was evaluated using the carrageenan-induced hind paw edema model in rats, following the method described by Yimer *et al.* [28]. Prior to the experiment, rats were fasted for 12 h with free access to water.

Animals were orally administered distilled water (control), crude extract (100, 200, or 400 mg/kg), the standard drug (10 mg/kg), or the isolated compound 4-(2ʺ-(4′-isopropylphenyl) propan-2ʺ-yl)-2,3-dihydrofuran (10, 20, and 40 mg/kg). Thirty minutes after treatment, 100 µL of carrageenan solution (1% w/v in normal saline) was injected subplantarly into the right hind paw of each rat to induce acute inflammation.

Paw edema was measured at 0, 1, 2, 3, 4, and 5 h after carrageenan injection using a water displacement plethysmometer, and the degree of inflammation was expressed in milliliters (mL). The early phase of inflammation (0–2 h) is primarily mediated by histamine and serotonin, whereas the late phase (3–5 h) is mainly associated with prostaglandin production.

The percentage inhibition of paw edema was calculated relative to the control group using the following formula. All behavioral observations and paw volume measurements were performed by an investigator blinded to treatment allocation.


$$\begin{array}{c} \%\text{ Ihnibition of paw edema}=\frac{(\text{Vt}-\text{Vo})\text{control}-(\text{Vt}-\text{Vo})\text{treated}}{(\text{Vt}-\text{Vo})\text{control}}*\text{10}\text{0} \end{array}$$

Where, Vt is the right hind paw thickness volume (in ml) at time t, and Vo is the right hind paw thickness volume (in ml) before carrageenan injection

### Statistical analysis

All the data obtained were expressed as mean ± SEM (standard error of the mean). One-way ANOVA was used in the statistical analysis of the data, and the Tukey Post Hoc Test for multiple comparisons was used to compare the results between groups. At a significance level of *p* < 0.05, the results were deemed statistically significant. The data were processed using SPSS version 27 software. We acknowledge that one-way ANOVA applied independently at each time point does not account for the within-subject correlation inherent in the time-course data (hot plate and carrageenan models); a repeated-measures ANOVA would be more appropriate for such designs and is noted as a statistical limitation of the current analysis. Additionally, statistical significance (p < 0.05) is reported as an indicator of reliability but should not be equated with biological or clinical relevance; effect sizes and 95% confidence intervals are presented where feasible and should be considered alongside p-values when interpreting magnitude of effect.

## Result and discussion

### Acute toxicity

The acute oral toxicity study revealed that administration of 2000 mg/kg of the *G. schweinfurthii* root extract did not cause any mortality in mice during the first 24 h or throughout the 14-day observation period. No apparent signs of toxicity, including lethargy, tremor, fatigue, paralysis, autonomic disturbances, or behavioral abnormalities, were observed in the treated animals. According to the OECD Guideline 423 limit test, the absence of mortality at 2000 mg/kg suggests that the median lethal dose (LD_50_) is greater than 2000 mg/kg. However, this result represents a limit test rather than a precise LD_50_ determination.

### Preliminary phytochemical screening

Qualitative analysis of the 80% methanolic root extract of *G. schweinfurthii* revealed the presence of tannins, terpenoids, flavonoids, saponins, and steroids, whereas tests for cardiac glycosides and anthraquinones yielded negative results.

### TLC analysis of 4-(2’‘-(4’-isopropylphenyl) propan-2’‘-yl)-2,3 dihydrofuran

Further phytochemical investigation of the root extracts of *G. schweinfurthii* over silica gel column chromatography led to the isolation of dihydrofuran. The TLC chromatogram of 4-(2’‘-(4’-isopropylphenyl) propan-2’‘-yl)-2,3 dihydrofuran showed Rf values of 0.5 in chloroform: methanol (6:1) and 0.35 in chloroform: ethyl acetate (2:1), both visualized under UV light at 254 nm and 366 nm.

The chromatographic purity of AL-03 was confirmed by TLC analysis using the two solvent systems (CHCl_3_: MeOH 6:1 and CHCl_3_: EtOAc 2:1), which consistently produced a single spot under UV detection. Repeated TLC analyses (n = 3) yielded identical Rf values, confirming the reproducibility and purity of the isolated compound, with no additional spots observed.

### Structural elucidation 4-(2’‘-(4’-isopropylphenyl) propan-2’‘-yl)-2,3 dihydrofuran (AL −03)

Compound AL-03 was isolated as a white amorphous powder with an Rf value of 0.50 in CHCl_3_: MeOH (6:1).

The ^1^H NMR spectrum (400 MHz, CDCl_3_) revealed two adjacent methylene groups at δ 4.60 (2H, t, J = 8.0 Hz, H-2) and δ 4.44 (2H, t, J = 8.0 Hz, H-3). Two sets of equivalent aromatic protons were observed at δ 7.59 (2H, d, J = 8.56 Hz, H-2′/H-6′) and δ 7.37 (2H, d, J = 8.56 Hz, H-3′/H-5′), forming an AA′BB′ spin system characteristic of a para-disubstituted benzene ring. A singlet at δ 7.80 (1H, s, H-5) corresponded to the proton of the dihydrofuran ring.

Signals characteristic of an isopropyl substituent was also observed, including δ 2.47 (6H, d, J ≈ 6.8 Hz, CH_3_) corresponding to two equivalent methyl groups and δ 1.90 (1H, m, CH) attributable to the methine proton. In addition, a singlet at δ 1.32 (6H, s) indicated the presence of two equivalent methyl groups. **The complete** ^**1**^**H NMR,** ^**13**^**C NMR, and HSQC spectra are provided in the Supporting Information (S4–S6 Figs in** **S1 File****).**

The ^13^C-NMR spectral data of AL-03 was in a good agreement with the findings from the ^1^H-NMR spectrum. The ^13^C NMR spectrum (100 MHz, CDCl_3_) revealed sixteen carbon signals, consistent with the molecular structure. Analysis of the ^13^C NMR and HSQC spectra indicated the presence of four methyl carbons [δ 12.73 (2×), 20.20 (2×)], two methylene carbons [δ 67.80, 44.93], six methine carbons [δ 29.34, 127.35 (2×), 129.86 (2×), 131.41], and four quaternary carbons [δ 31.58, 131.91, 145.72, 151.52].

**Fig 1:**
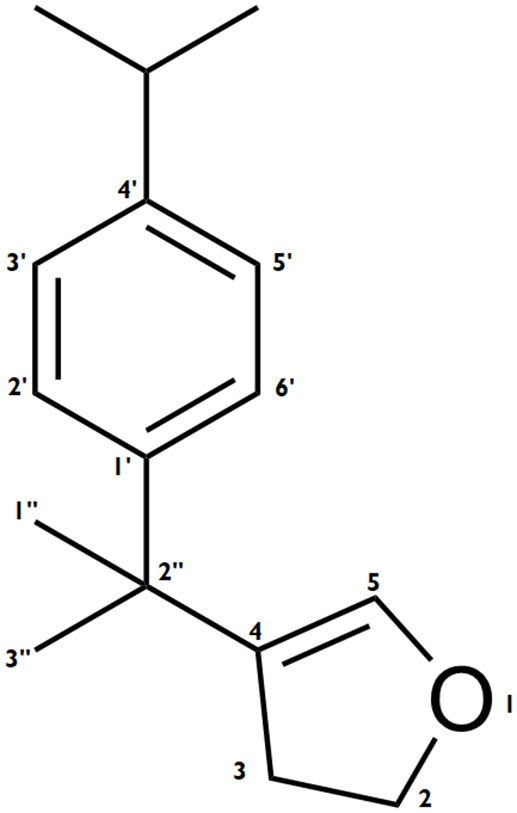
The compound AL-03 elucidated as 4-(2''-(4'-isopropylphenyl) propan-2''-yl)-2,3 dihydrofuran.

The combined interpretation of ^1^H NMR, ^13^C NMR, and HSQC spectral data confirmed the structure of the isolated compound as 4-(2ʺ-(4′-isopropylphenyl) propan-2ʺ-yl)-2,3-dihydrofuran (Fig 1). A complete list of proton and carbon assignments is provided in Table 1, and **the full NMR spectra are presented in Supporting Information.**

**Table 1:** ^1^H NMR and ^13^C NMR chemical shifts for dihydrofuran (AL-03).

C/H numbering	^1^H (δ, ppm)	^13^C (δ, ppm)
2	4.60 *t* (*J* = 8.0 Hz)	67.8
3	4.44 *t* (*J* = 8.0 Hz)	44.93
5	7.80 *s*	131.12
4	–	131.91
1’	–	151.57
4’	–	145.72
2’ & 6’	7.59 *d* (*J* = 8.5 Hz)	127.35
3’ & 5’	7.37 *d* (*J* = 8.5 Hz)	129.86
1’‘ & 3’‘	1.32 *s*	29.53
2’‘	–	32.23
2CH_3_ (Isopropyl)	2.47 *d*	12.73, 20.20
CH (Isopropyl)	1.9 *s*	30.03

Note: s = singlet, d = doublet, t = triplet, m = multiplet.

### Analgesic activity

#### Analgesic activity of root extract using acetic acid-induced writhing method.

This model is widely employed for assessing the peripheral analgesic potential of natural products because of its high sensitivity [28]. Intraperitoneal stimulation triggers the release of inflammatory mediators, including histamine, serotonin, and bradykinin, which activate sensory nerve endings. Moreover, elevated concentrations of PGE_2_, PGF_2_, and lipoxygenase-derived metabolites have been detected in the peritoneal fluid during this assay [7]. The writhing response is typically manifested by abdominal muscle contractions, forelimb extension, and body elongation.

In this study, the root extract of *G. schweinfurthii* demonstrated a significant reduction (*p* < 0.001) in the number of writhes in mice at doses of 200 and 400 mg/kg relative to the negative control. Furthermore, the 400 mg/kg dose elicited a significantly greater reduction (p < 0.001) in writhing frequency compared to the 100 and 200 mg/kg doses (**Table 2**). The 100 mg/kg dose reduced writhing by 13.9%; however, this reduction was not statistically significant compared with the negative control (p > 0.05). Similarly, acetylsalicylic acid caused a significant decrease in writhing frequency (p < 0.001) relative to the 100 and 200 mg/kg extract doses, but its effect did not differ significantly from that of the 400 mg/kg extract dose (**Table 2**). The present findings are consistent with a previous report on *G. asiatica*, where the 80% methanolic extract exhibited significant analgesic activity at a dose of 400 mg/kg. Notably, at lower doses, the extract of *G. schweinfurthii* demonstrated comparatively greater peripheral analgesic activity than that reported for *G. asiatica* [11]. The analgesic effect observed for *G. schweinfurthii* in this study may be linked to its phytochemical constituents, including tannins, saponins, terpenoids, flavonoids, and steroids, which are known to reduce prostaglandin synthesis through inhibition of the cyclooxygenase and/or lipoxygenase pathways [1].

**Table 2:** Effect of 80% methanolic root extracts of *G. schweinfurthii* on acetic acid induced writhing in mice.

Group	Number of Writhings (Mean ± SEM) (95% CI)	% of Inhibition
DW	25.17 ± 1.815 (20.50–29.84)	
Aspirin (150 mg/ kg)	4.67 ± 0.422^a1b1c1^ (3.59–5.75)	80.77
GS (100 mg/ kg)	21.67 ± 0.76 (19.72–23.62)	13.90
GS (200 mg/ kg)	10.87 ± 0.477^a1b1^ (9.64–12.10)	56.81
GS (400 mg/ kg)	6.17 ± 0.477^a1b1c2^ (4.94–7.40)	75.48

**Note**: Values are expressed as Mean ± SEM (95% CI) (N = 6); analysis was performed with One-Way ANOVA followed by Tukey post hoc test; ^a^ against the control, ^b^ against 100 mg, ^c^ against 200 mg; ^1^ P < 0.001, ^2^ P < 0.05. **Abbreviation**: DW = distilled water 10 ml/kg; GS100 = *G. schweinfurthii* extract 100 mg/kg; GS200 = *G. schweinfurthii* extract 200 mg/kg; GS400 = *G. schweinfurthii* extract 400 mg/kg.

#### Analgesic activity of root extract using hot plate method.

The hot plate assay was used to evaluate the central analgesic activity of *Grewia schweinfurthii* extract in mice. This method was selected because of its high sensitivity to potent analgesics, minimal tissue damage, reliable data accuracy, and shorter processing time [28]. In this test, mice are placed on a plate maintained at 55 °C, which induces two characteristic responses—paw licking and jumping—measured by their reaction times. Both behaviors are considered to be supraspinally mediated [29].

**Fig 2:**
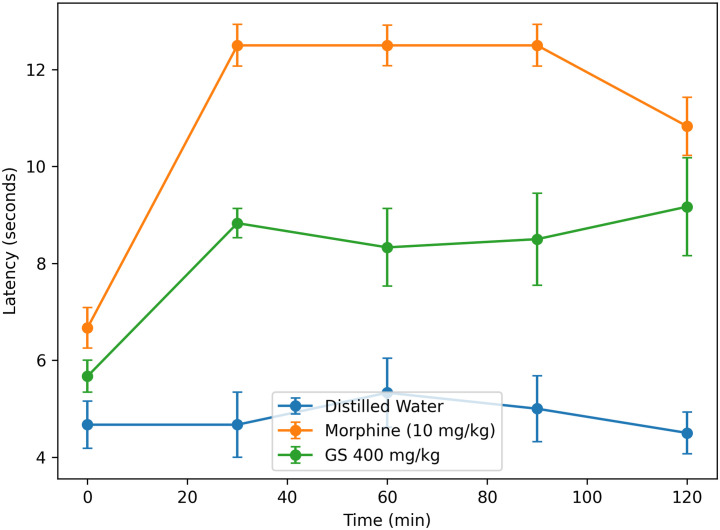
Time-course of central analgesic activity of *Grewia schweinfurthii* root extract in the hot plate test. Latency to paw licking or jumping was measured at 0, 30, 60, 90, and 120 minutes after oral administration of distilled water (10 mL/kg), *Grewia schweinfurthii* extract (400 mg/kg), or morphine (10 mg/kg). Data are expressed as mean ± SEM (n = 6 per group). Statistical analysis was performed using one-way ANOVA followed by Tukey’s post hoc test. Significant differences compared with the negative control were observed at multiple time points for morphine (p < 0.001) and for the extract at 400 mg/kg (p < 0.05).

The root extract at 400 mg/kg (p < 0.05) and morphine at 10 mg/kg (p < 0.001) exhibited significant analgesic effects at multiple time points (30, 60, 90, and 120 minutes) compared with the negative control (**Fig 2**). In contrast, the root extract at 100 mg/kg did not show any significant analgesic activity over the observation period (**Table 3**; **Fig 2**). Moreover, doses of 200 mg/kg and 400 mg/kg of the root extract significantly elevated the pain threshold by extending the reaction time, although it required up to 120 minutes to achieve the maximum effect for all doses. The enhanced activity observed at 400 mg/kg throughout the study period may be attributed to the higher concentration of active metabolites. These findings are consistent with a previous study on *G. asiatica*, where methanolic and aqueous extracts at 400 mg/kg significantly (p < 0.01, p < 0.05) increased reaction time [11]. The central analgesic effect of the extract is likely mediated through the inhibition of the synthesis of prostaglandins, leukotrienes, and other endogenous compounds involved in central pain transmission [28].

**Table 3:** Analgesic effect of 80% methanolic root extracts of *Grewia schweinfurthii* using hot plate method.

Group	Latency (Sec) ± SEM (95% CI)				
	0 min	30 min	60 min	90 min	120 min
DW (10 mL/kg)	4.67 ± 0.49 (3.41–5.93)	4.67 ± 0.67 (2.95–6.39)	5.33 ± 0.71 (3.50–7.16)	5.00 ± 0.68 (3.25–6.75)	4.50 ± 0.43 (3.39–5.61)
Mo (10 mg/kg)	6.67 ± 0.42 (5.59–7.75)	12.50 ± 0.43 ^a1^ (11.39–13.61)	12.50 ± 0.42 ^a1^ (11.42–13.58)	12.50 ± 0.43 ^a1^ (11.39–13.61)	10.83 ± 0.60 ^a1^ (9.29–12.37)
GS100 (100 mg/kg)	5.17 ± 0.30 (4.40–5.94)	5.83 ± 0.60 (4.29–7.37)	6.50 ± 0.43 (5.39–7.61)	6.00 ± 0.57 (4.53–7.47)	6.00 ± 0.36 (5.06–6.94)
GS200 (200 mg/kg)	5.20 ± 0.30 (4.43–5.97)	7.33 ± 0.33 ^a2^ (6.48–8.18)	6.50 ± 0.76 ^a2^ (4.55–8.45)	8.00 ± 0.57 ^a2^ (6.53–9.47)	6.83 ± 0.60 (5.29–8.37)
GS400 (400 mg/kg)	5.67 ± 0.33 (4.82–6.52)	8.83 ± 0.30 ^a2^ (8.06–9.60)	8.33 ± 0.80 ^a2^ (6.27–10.39)	8.50 ± 0.95 ^a2^ (6.06–10.94)	9.17 ± 1.01 ^a2^ (6.57–11.77)

**Note**: The value expressed as Mean ± SEM (95% CI) (N=6); analysis was performed with One-Way ANOVA followed by Tukey post hoc test; ^a^ against the control, P<0.001, ^2^ P<0.05. **Abbreviation**: DW- distilled water 10ml/kg; MO (10 mg/kg) = morphine 10mg/kg; GS100 = *G. schweinfurthii* extract 100 mg/kg; GS200 = *G. schweinfurthii* extract 200 mg/kg; GS400 = *G. schweinfurthii* extract 400 mg/kg.

### Anti-inflammatory activities

#### Anti-inflammatory activities of root extract.

The anti-inflammatory potential of the root extract of *G. schweinfurthii* in the acute phase of inflammation was evaluated using carrageenan-induced paw edema. This model is widely employed to assess the anti-inflammatory activity of drugs and to investigate the underlying mechanisms of inflammation. It serves as an appropriate *in vivo* model for studying the anti-inflammatory effects of natural products due to its involvement with multiple inflammatory mediators [29].

Acute inflammation (edema) was induced by subplantar injection of carrageenan (1% v/v in normal saline) into the left hind paw of rats. Following carrageenan administration, local acute inflammation occurs due to the sequential release of various endogenous inflammatory mediators, which are released in a biphasic manner. The initial phase (0–2.5 hours) is primarily mediated by histamine, serotonin, and bradykinin. The late phase (2.5–5 hours) is sustained by overproduction of COX-2 and its proinflammatory prostaglandins, along with infiltration of polymorphonuclear leukocytes (neutrophils). Additionally, oxygen-derived free radicals such as nitric oxide (NO), superoxide anions (O_2_^−^), and hydroxyl radicals (OH^−^) are released, playing a critical role in both the initiation and progression of acute inflammation [27,28].

**Fig 3:**
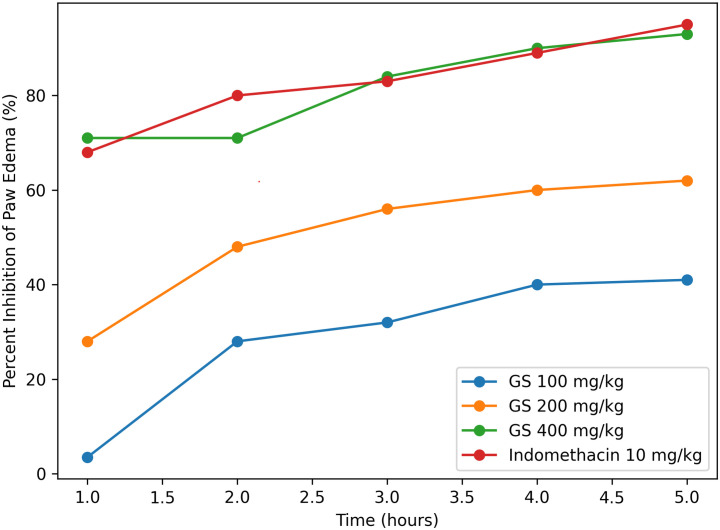
Time-course of anti-inflammatory activity of *Grewia schweinfurthii* root extract in carrageenan-induced paw edema. Percent inhibition of paw edema was measured at 1, 2-, 3-, 4-, and 5-hours following carrageenan injection. Rats were treated orally with distilled water (10 mL/kg), *Grewia schweinfurthii* extract (100, 200, or 400 mg/kg), or indomethacin (10 mg/kg). Data are presented as mean percent inhibition relative to control (n = 6 per group). Statistical analysis was conducted using one-way ANOVA followed by Tukey’s post hoc test. Significant inhibition was observed at later time points, particularly at 4–5 hours (p < 0.05–0.001).

The time-course anti-inflammatory activity of the extract in carrageenan-induced paw edema is presented in (**Fig 3**). At the fifth observation time point, all doses of the extract produced the highest percentage inhibition of edema, with values of 41%, 62%, and 93% (**Table 4**). These findings indicate that the anti-inflammatory effect of the extract is dose-dependent and highly effective in suppressing prostaglandin release. Notably, at this time point, the edema inhibitory effect of the highest extract dose (400 mg/kg) was comparable to that of the reference drug, indomethacin (10 mg/kg), with inhibition values of 93.0% and 95%, respectively (**Table 4**). These findings are consistent with previous studies on *G. asiatica*, where both methanolic and aqueous extracts produced significant, dose-dependent reductions in paw edema during both the early and late phases of inflammation (11). The anti-inflammatory effect is likely mediated through inhibition of inflammatory mediators involved in carrageenan-induced edema, including histamine, serotonin, kinins, and prostaglandins, as well as reactive oxygen species such as nitric oxide, hydroxyl radicals, and superoxide anions [30]. Regarding the biphasic nature of carrageenan-induced inflammation, the data in Table 4 show that the extract was more effective during the late phase (3–5 h), which is predominantly driven by prostaglandin overproduction via COX-2. Inhibition values at 1–2 h (early phase, mediated by histamine and serotonin) were notably lower across all doses compared with those at 3–5 h, suggesting that the extract’s primary mechanism of action is more closely aligned with prostaglandin pathway suppression than with histamine or serotonin antagonism. The 93% inhibition at 400 mg/kg at 5 h, while numerically close to indomethacin (95%), should be interpreted cautiously: the extract was used at 400 mg/kg compared to indomethacin at 10 mg/kg, and the extract has not been standardized. This dose difference of 40-fold, combined with the absence of extract standardization or quantification of active constituents, precludes a direct potency equivalence claim. The high percent-inhibition figures are consistent with the validated sensitivity of the carrageenan model and are reported in the context of efficacy demonstration rather than clinical equivalence.

**Table 4:** Anti-inflammatory effect of 80% methanolic root extract *Grewia schweinfurthii* using carrageenan-induced paw edema in rat.

Treatment (mg/kg)	Edema Volume (mL)/ Percent Edema Inhibition					
	Baseline	1 h	2 h	3 h	4 h	5 h
DW (10 mL/kg)	1.09 ± 0.03 (1.01–1.17)	1.37 ± 0.05 (1.24–1.50)	1.44 ± 0.05 (1.31–1.57)	1.55 ± 0.09 (1.32–1.78)	1.62 ± 0.09 (1.39–1.85)	1.67 ± 0.06 (1.52–1.82)
Indo (10)	1.07 ± 0.01 (1.04–1.10)	1.16 ± 0.03 ^a1^ (68%) (1.08–1.24)	1.14 ± 0.02 ^a1^ (80%) (1.09–1.19)	1.15 ± 0.03 ^a1^ (83%) (1.07–1.23)	1.13 ± 0.01 ^a1^ (89%) (1.10–1.16)	1.11 ± 0.01 ^a1^ (95%) (1.08–1.14)
GS100	1.05 ± 0.03 (0.97–1.13)	1.32 ± 0.03 (3.5%) (1.24–1.40)	1.30 ± 0.05 (28%) (1.17–1.43)	1.36 ± 0.01 ^a2^ (32%) (1.33–1.39)	1.37 ± 0.02 ^a2^ (40%) (1.32–1.42)	1.39 ± 0.01 ^a1 (^41%) (1.36–1.42)
GS200	1.06 ± 0.03 (0.98–1.14)	1.26 ± 0.03 (28%) (1.18–1.34)	1.24 ± 0.02 ^a2^ (48%) (1.19–1.29)	1.26 ± 0.00 ^a2^ (56%) (1.26–1.26)	1.27 ± 0.01 ^a1^ (60%) (1.24–1.30)	1.28 ± 0.00 ^a1^ (62%) (1.28–1.28)
GS400	1.10 ± 0.02 (1.05–1.15)	1.18 ± 0.01^a2^ (71%) (1.15–1.21)	1.18 ± 0.01 ^a1^ (71%) (1.15–1.21)	1.17 ± 0.03 ^a1^ (84%) (1.09–1.25)	1.15 ± 0.01 ^a1^ (90%) (1.12–1.18)	1.14 ± 0.03 ^a1^ (93%) (1.06–1.22)

**Note**: Values are expressed as Mean ± SEM (95% CI) (N=6); analysis was performed with One-Way ANOVA followed by Tukey post hoc test; ^a^ against the control, ^1^P<0.001, ^2^ P<0.05. **Abbreviation**: DW- distilled water 10 ml/kg; INDO = indomethacin 10 mg/kg; GS100 = *G. schweinfurthii* 100 mg/kg; GS200 = *G. schweinfurthii* 200 mg/kg; GS400 = *G. schweinfurthii* 400 mg/kg.

#### Anti-inflammatory activity of 4-(2’‘-(4’-isopropylphenyl) propan-2’‘-yl)-2,3-dihydrofuran.

The anti-inflammatory activity of the isolated compound was evaluated using carrageenan-induced paw edema in rats during the acute phase of inflammation. From the first hour through to 5 hours, both the standard drug and the 40 mg/kg dose of the isolated compound showed a statistically significant inhibition of paw edema (p < 0.001 and p < 0.05, respectively) compared with the negative control. At the 4th and 5th hour measurements, all doses of the isolated compound (10, 20, and 40 mg/kg), as well as the standard drug, demonstrated a significant inhibitory effect (p < 0.001) relative to the negative control (**Table 5).**

**Table 5:** Anti-inflammatory effect of 4-(2’‘-(4’-isopropylphenyl) propan-2’‘-yl)-2,3-dihydrofuran isolated from *Grewia schweinfurthii* on carrageenan-induced paw edema in rat.

Treatment (mg/kg)	Edema Volume (mL)/ Percent Edema Inhibition					
	Baseline	1 h	2 h	3 h	4 h	5 h
DW (10 mL/kg)	1.09 ± 0.03 (1.01–1.17)	1.37 ± 0.05 (1.24–1.50)	1.44 ± 0.05 (1.31–1.57)	1.55 ± 0.09 (1.32–1.78)	1.62 ± 0.09 (1.39–1.85)	1.67 ± 0.06 (1.52–1.82)
Indo (10)	1.07 ± 0.00 (1.07–1.07)	1.16 ± 0.03 ^a1^ (68%) (1.08–1.24)	1.14 ± 0.02 ^a1^ (80%) (1.09–1.19)	1.15 ± 0.03 ^a1^ (82%) (1.07–1.23)	1.13 ± 0.01 ^a1^ (88%) (1.10–1.16)	1.11 ± 0.01 ^a1^ (95%) (1.08–1.14)
CX10mg	1.05 ± 0.02 (1.00–1.10)	1.29 ± 0.02(31%) (1.24–1.34)	1.23 ± 0.02 ^a2^ (35%) (1.18–1.28)	1.28 ± 0.02 ^a2^ (50%) (1.23–1.33)	1.29 ± 0.03 ^a1^ (54%) (1.21–1.37)	1.28 ± 0.04 ^a1^ (60%) (1.18–1.38)
CX20mg	1.06 ± 0.02 (1.01–1.11)	1.27 ± 0.01(40%) (1.24–1.30)	1.21 ± 0.02 ^a2^ (46%) (1.16–1.26)	1.26 ± 0.02 ^a2^ (56%) (1.21–1.31)	1.27 ± 0.02 ^a1^ (60%) (1.22–1.32)	1.23 ± 0.03 ^a1^ (70%) (1.15–1.31)
CX40mg	1.06 ± 0.02 (1.01–1.11)	1.25 ± 0.01 ^a2^ (45%) (1.22–1.28)	1.20 ± 0.01 ^a1^ (50%) (1.17–1.23)	1.24 ± 0.01 ^a1^ (61%) (1.21–1.27)	1.25 ± 0.02 ^a1^ (64%) (1.20–1.30)	1.21 ± 0.03 ^a1^ (75%) (1.13–1.29)

**Note**: The value expressed as Mean ± SEM (N = 6); analysis was performed with One-Way ANOVA followed by Tukey post hoc test; ^a^ against the control, ^1^P < 0.001, ^2^ P < 0.05. **Abbreviation**: DW- distilled water 10 ml/kg; INDO = indomethacin 10 mg/kg; CX10mg = AL-03 10 mg/kg; CX20mg = AL-03 20 mg/kg; CX40mg = AL-03 40 mg/kg.

**Fig 4:**
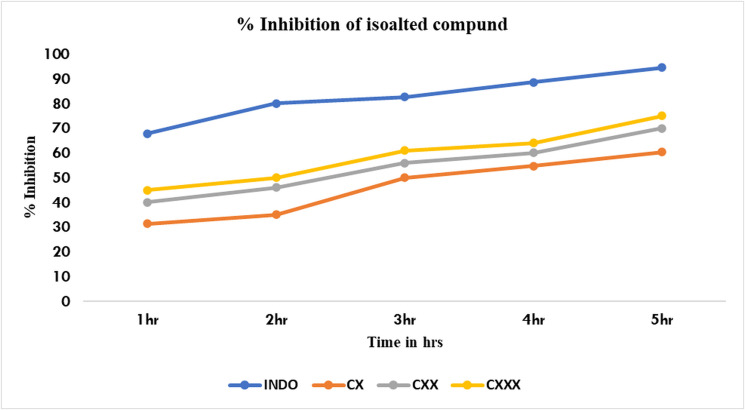
Comparison of percentage suppression of paw edema induced by carrageenan of the isolated compound at different doses CX (10 mg/kg), CX (20 mg/kg) and CX (40 mg/kg) with that of the standard drug indomethacin (10 mg/kg).

As shown in Fig 4, the maximum and minimum volume reductions were achieved at all doses of the isolated compound and the standard solution at the 5^th^ and 1^st^ h, respectively, of the study period. At the peak of activity (5 h), the percent inhibition for (10, 20, 40 mg/kg) was 60.3%, 70.6% and 75%, respectively. However, the inhibition for the standard drug was 94.5%.

#### Cohesive integration of peripheral, central, and anti-inflammatory effects.

The extract demonstrated significant peripheral analgesic activity in the acetic acid-induced writhing model, particularly at 200 and 400 mg/kg, indicating potential inhibition of inflammatory mediator release. In contrast, the hot plate assay revealed central analgesic activity at higher doses, suggesting possible supraspinal involvement. Importantly, the anti-inflammatory activity observed in the carrageenan model—especially during the late phase (3–5 h)—supports the peripheral analgesic findings. The marked suppression of paw edema during the prostaglandin-dependent phase suggests that the extract may plausibly interfere with inflammatory mediator pathways, although direct COX inhibition was not assessed in this study.

The isolated compound AL-03 also demonstrated dose-dependent inhibition of paw edema, achieving 75% inhibition at 40 mg/kg at 5 h, supporting its contribution to the extract’s pharmacological activity. Phytochemical screening revealed the presence of flavonoids, terpenoids, steroids, tannins, and saponins. These classes of compounds are widely reported to exhibit anti-inflammatory and analgesic activities in the literature, possibly through modulation of prostaglandin synthesis, nitric oxide production, and oxidative stress pathways. However, mechanistic confirmation requires further biochemical investigation. More specifically, the flavonoids and terpenoids detected in the extract are well-documented inhibitors of COX and lipoxygenase (LOX) pathways, which is mechanistically consistent with the marked inhibition of carrageenan-induced edema observed during the prostaglandin-dependent late phase (3–5 h). Tannins and saponins, also present in the extract, have been reported to suppress the release of histamine, serotonin, and bradykinin — mediators that predominate during the early phase of inflammation — which may explain the inhibition observed at 1–2 h in the current study. Regarding the isolated compound AL-03, the dihydrofuran scaffold bearing a para-isopropyl phenyl group is structurally consistent with anti-inflammatory activity; dihydrofuran-containing terpenoid derivatives have been reported to modulate prostaglandin pathways and suppress COX-2 expression in prior literature. Taken together, the phytochemical profile of the extract and the structural features of AL-03 provide a plausible chemical basis for the observed pharmacological activities, though direct enzyme inhibition assays will be required to confirm specific mechanistic targets.

Compared to morphine, the extract exhibited moderate central analgesic activity, with maximal latency increases significantly lower than morphine (p < 0.001), suggesting a weaker but measurable central component.

## Conclusion

The 80% methanolic root extract of *G. schweinfurthii* demonstrated significant antinociceptive effects in both central (hot plate) and peripheral (acetic acid–induced writhing) rodent models, as well as potent anti-inflammatory activity in the carrageenan-induced paw edema model. The isolated compound AL-03 (4-(2ʺ-(4′-isopropylphenyl) propan-2ʺ-yl)-2,3-dihydrofuran) also exhibited dose-dependent inhibition of paw edema, supporting its potential contribution to the extract’s activity. These results are based on animal studies and should not be directly extrapolated to humans. These studies include the use of acute models only, absence of mechanistic investigations, and lack of comprehensive toxicity and pharmacokinetic data. Future studies should evaluate the isolated compound independently, explore molecular mechanisms, assess safety, and investigate chronic models to better define translational relevance. Importantly, the simultaneous evaluation of both a crude extract and a structurally characterized isolated compound within a single study provides a scientifically rigorous framework for linking observed pharmacological effects to specific chemical entities. This dual extract–compound approach represents a key methodological strength of this work and constitutes a meaningful contribution to the growing literature on African medicinal plants.

### Limitations

Several methodological and interpretative limitations of the present study should be acknowledged. First, only acute rodent models were employed; chronic or subacute models were not investigated, limiting conclusions regarding long-term efficacy and safety. In addition, no pharmacokinetic, bioavailability, or sub-chronic toxicity studies were performed, and therefore translational interpretation remains preliminary.

Second, phytochemical characterization was limited. The extract was evaluated qualitatively, but quantitative profiling of major metabolite classes, including total phenolics and flavonoids, was not performed, limiting precise correlation between phytochemical composition and pharmacological activity. Furthermore, structural elucidation of AL-03 relied primarily on 1D and 2D NMR spectroscopy, and confirmatory high-resolution mass spectrometric analyses (HRMS or LC-MS/MS) were not conducted.

Third, mechanistic investigations were limited because no direct biochemical assays, such as COX-1/2 inhibition, nitric oxide quantification, or cytokine analysis, were performed. Consequently, mechanistic interpretations are based on indirect pharmacological evidence and previously reported literature rather than direct molecular measurements.

Finally, several experimental and statistical limitations should be considered. Sample size (n = 6 per group) was determined based on established practice in comparable pharmacological studies rather than formal a priori power analysis, which may have reduced sensitivity for detecting smaller effect sizes. Animals of both sexes were included without sex-stratified analysis, and potential sex-dependent differences in nociceptive and inflammatory responses were therefore not evaluated. In addition, one-way ANOVA was applied separately at individual time points for repeated-measure experiments, representing a statistical limitation for the hot plate and carrageenan-induced paw edema models. Moreover, because extract standardization was not performed, direct potency comparisons with reference drugs should be interpreted cautiously.

## Supporting information

S1 File Supporting Information. This file contains S1–S6 Figures, including TLC chromatograms and NMR spectra of AL-03.
(DOCX)

S2 File PLOS ONE Humane Endpoints Checklist. (DOCX)
