# Supplementary material for: Evaluation of analgesic and anti-inflammatory activities of the root extract of Grewia schweinfurthii Burret and its major chemical constituents
Source: PLoS One. 2026 Jul 22;21(7):e0353400. doi: 10.1371/journal.pone.0353400 (PMC13483540; doi:10.1371/journal.pone.0353400)
Supplement: Supplementary file 1 [file pone.0353400.s001.docx]

Supplementary Material

**S1 Fig.** TLC chromatogram of AL-03 (4-(2''-(4'-isopropylphenyl)propan-2''-yl)-2,3-dihydrofuran) using CHCl₃:MeOH (6:1) as the solvent system. Plates were visualized under UV light at 254 nm.

**S2 Fig.** TLC chromatogram of AL-03 using CHCl₃:MeOH (6:1) as the solvent system, visualized under UV light at 366 nm.

**S3 Fig.** TLC chromatogram of the crude methanolic root extract of G. schweinfurthii using CHCl₃:MeOH (6:1) as the solvent system and visualized under UV light at 254 nm.

**S4 Fig.** ¹H NMR spectrum of AL-03 in CDCl₃ at 400 MHz. Chemical shifts (δ, ppm) and signal multiplicities (s = singlet, d = doublet, t = triplet, q = quartet, m = multiplet) are indicated.

**S5 Fig.** ¹³C NMR spectrum of AL-03 in CDCl₃ at 100 MHz. Chemical shifts (δ, ppm) for all carbon atoms are shown.

**S6 Fig.** HSQC spectrum of AL-03 showing correlations between protons and carbons to support structural assignment. Recorded in CDCl₃.

***S1 Fig.*** *TLC chromatogram of AL-03 (4-(2''-(4'-isopropylphenyl)propan-2''-yl)-2,3-dihydrofuran) using CHCl₃:MeOH (6:1) as the solvent system. Plates were visualized under UV light at 254 nm.*

***S2 Fig.*** *TLC chromatogram of AL-03 using CHCl₃:MeOH (6:1) as the solvent system, visualized under UV light at 366 nm.*

***S3 Fig.*** *TLC chromatogram of the crude methanolic root extract of G. schweinfurthii using CHCl₃:MeOH (6:1) as the solvent system and visualized under UV light at 254 nm.*

*^S4 Fig. ¹H NMR spectrum of AL-03 in CDCl₃ at 400 MHz. Chemical shifts (δ, ppm) and signal multiplicities (s = singlet, d = doublet, t = triplet, q = quartet, m = multiplet) are indicated.^*

***S5 Fig.*** *¹³C NMR spectrum of AL-03 in CDCl₃ at 100 MHz. Chemical shifts (δ, ppm) for all carbon atoms are shown.*

***S6 Fig.*** *HSQC spectrum of AL-03 showing correlations between protons and carbons to support structural assignment. Recorded in CDCl₃.*

*^S4 Fig. ¹H NMR spectrum of AL-03 in CDCl₃ at 400 MHz. Chemical shifts (δ, ppm) and signal multiplicities (s = singlet, d = doublet, t = triplet, q = quartet, m = multiplet) are indicated.^*

***S5 Fig.*** *¹³C NMR spectrum of AL-03 in CDCl₃ at 100 MHz. Chemical shifts (δ, ppm) for all carbon atoms are shown.*

***S6 Fig.*** *HSQC spectrum of AL-03 showing correlations between protons and carbons to support structural assignment. Recorded in CDCl₃.*
