# Supplementary material for: Evaluation of analgesic and anti-inflammatory activities of the root extract of Grewia schweinfurthii Burret and its major chemical constituents
Source: PLoS One. 2026 Jul 22;21(7):e0353400. doi: 10.1371/journal.pone.0353400 (PMC13483540; doi:10.1371/journal.pone.0353400)
Supplement: Supplementary file 2 [file pone.0353400.s002.docx]

*PLOS ONE* Humane Endpoints Checklist

*PLOS ONE* manuscript number: PONE-D-26-01189

**Complete the following if your study design includes death of a regulated animal as a likely outcome or planned experimental endpoint. Please also include all information in the Methods section of your manuscript.**

**ITEM 1.** **Describe whether humane endpoints* were used for all animals involved in the study.**

|  | **Recommendation** | **Section/Paragraph** |
| --- | --- | --- |
| **If humane endpoints* were used, report the following:** | | |
| **1** | **The specific criteria used to determine when animals should be euthanized** | Animals were humanely euthanized if they exhibited severe or persistent signs of pain, distress, or toxicity, including marked body weight loss (>20%), lethargy, inability to access food or water, severe diarrhea, tremors, paralysis, abnormal posture, or any other indication of poor prognosis or compromised welfare.  **Section/Paragraph:** Methods → *Animal care, ethical approval, and humane endpoints* |
| **2** | **Once animals reached endpoint criteria, the amount of time elapsed before euthanasia** | Animals meeting predefined humane endpoint criteria were euthanized immediately upon identification of severe or irreversible distress to prevent unnecessary suffering.  **Section/Paragraph:** Methods → *Animal care, ethical approval, and humane endpoints* |
| **3** | **Whether any animals died before meeting criteria for euthanasia** | No animals died before meeting humane endpoint criteria. No animals were allowed to die as an experimental endpoint, and no unexpected mortality occurred during the study.  **Section/Paragraph:** Methods → *Animal care, ethical approval, and humane endpoints* |
| **If humane endpoints* were not used, report the following:** | | |
| **1** | **A scientific and ethical justification for the study design, including the reasons why humane endpoints could not be used, and discussion of alternatives that were considered but could not be used** |  |
| **2** | **Whether the institutional animal ethics committee specifically reviewed and approved the anticipated mortality in the study design** |  |

**ITEM 2.** **Include the following details of the study design and outcomes.**

|  | **Recommendation** | **Section/Paragraph** |
| --- | --- | --- |
| **1** | **The duration of the experiment** | The acute oral toxicity study included continuous monitoring during the first 24 hours, followed by daily observations for 14 days. Analgesic and anti-inflammatory experiments were conducted over defined short-term periods in accordance with established protocols.  **Section/Paragraph:** Methods → *Acute oral toxicity test*; *Evaluation of analgesic activity*; *Anti-inflammatory activity* |
| **2** | **The numbers of animals used, euthanized, and found dead (if any); the cause of death for all animals** | Animals were randomly allocated into experimental groups of six per group, as described in the Methods section. No animals were found dead during the study. Euthanasia was performed only when required by predefined humane endpoints; no deaths were attributable to experimental procedures.  **Section/Paragraph:** Methods → *Animal grouping and dosing*; *Animal care, ethical approval, and humane endpoints* |
| **3** | **How frequently animal health and behavior were monitored** | Animals were monitored at least twice daily for general health and behavior throughout the study period, with more frequent observations during the acute toxicity phase and immediately following experimental interventions.  **Section/Paragraph:** Methods → *Animal care, ethical approval, and humane endpoints* |
| **4** | **All animal welfare considerations taken, including efforts to minimize suffering and distress, use of analgesics or anaesthetics, or special housing conditions** | All efforts were made to minimize pain, suffering, and distress. Animals were housed under standard laboratory conditions with ad libitum access to food and water and allowed a one-week acclimatization period prior to experimentation. Cut-off times were applied in nociceptive assays to prevent tissue damage, and the number of animals used was minimized in accordance with the principles of Replacement, Reduction, and Refinement (3Rs).  **Section/Paragraph:** Methods → *Experimental animals*; *Animal care, ethical approval, and humane endpoints* |
| **5** | **Any special training in animal care or handling provided for research staff** | All experimental procedures were performed by trained personnel experienced in laboratory animal handling and in vivo pharmacological assays, in accordance with institutional and international ethical guidelines.  **Section/Paragraph:** Methods → *Animal care, ethical approval, and humane endpoints* |

***Definition of a humane endpoint**

A humane endpoint is an experimental endpoint at which animals are euthanized when they display early markers associated with death or poor prognosis of quality of life, or specific signs of severe suffering or distress. Humane endpoints are used as an alternative to allowing such conditions to continue or progress to death following the experimental intervention (“death as an endpoint”), or only euthanizing animals at the end of an experiment. Before a study begins, researchers define the practical observations or measurements that will be used during the study to recognize a humane endpoint, based on anticipated clinical, physiological, and behavioral signs. These may include, for instance, body temperature or weight changes, tumor size or appearance, abnormal behaviors, pathological changes, ruffled fur, reduced mobility, body posture, or expression of specific body fluid markers. Please see the NC3Rs guidelines for more information.

**ARRIVE Guidelines**

*PLOS ONE* encourages authors to follow the [Animal Research: Reporting of In Vivo Experiments (ARRIVE) guidelines](http://www.nc3rs.org.uk/arrive-guidelines) for all submissions describing laboratory-based animal research and to upload a completed [ARRIVE Guidelines Checklist](http://www.nc3rs.org.uk/sites/default/files/documents/Guidelines/NC3Rs%20ARRIVE%20Guidelines%20Checklist%20%28fillable%29.pdf) to be published as supporting information.
