# Supplementary material for: Evaluation of analgesic and anti-inflammatory activities of the root extract of Grewia schweinfurthii Burret and its major chemical constituents
Source: PLoS One. 2026 Jul 22;21(7):e0353400. doi: 10.1371/journal.pone.0353400 (PMC13483540; doi:10.1371/journal.pone.0353400)
Supplement: Supplementary file 3 [file pone.0353400.s003.docx]

**Response to Reviewers**

**Manuscript Title:** Evaluation of analgesic and anti-inflammatory activities of the root extract of *Grewia schweinfurthii* Burret and characterization of its major chemical constituent
**Manuscript Number:** PONE-D-26-01189

We sincerely thank the Editor and the Reviewers for their careful evaluation of our manuscript and for the constructive comments and suggestions. We have revised the manuscript thoroughly in response to all comments. The revisions have improved the clarity, methodological transparency, and scientific rigor of the manuscript. All changes have been incorporated into the revised manuscript and highlighted where appropriate.

Below we provide a **point-by-point response** to each comment.

**Editor Comments**

**Comment 1**

Please ensure that your manuscript meets PLOS ONE style requirements, including file naming.

**Response:**
Thank you for this guidance. The manuscript has been carefully revised to comply with PLOS ONE formatting and style requirements, including file naming conventions.

**Changes made:**

Manuscript formatting updated throughout.

**Comment 2**

Provide additional information regarding animal experiments including sacrifice methods, anesthesia, analgesia, and efforts to alleviate suffering.

**Response:**
We appreciate this important comment. Additional details regarding animal handling, anesthesia procedures, humane endpoints, and sacrifice methods have now been included in the Methods section. The revised text clarifies the ethical considerations and measures taken to minimize animal suffering in accordance with institutional ethical guidelines.

**Changes made:**

Methods section – Animal experimentation subsection.

**Comment 3**

Provide additional NMR spectroscopy details.

**Response:**
We thank the Editor for this valuable suggestion and agree that providing detailed spectroscopic information improves the transparency and reproducibility of compound identification. In the revised manuscript, the Methods section has been expanded to include additional details regarding the NMR analysis, including the instrument model, magnetic field strength, nuclei measured, solvent system, reference standard, and experimental conditions used during spectral acquisition.

The structural elucidation of the isolated compound was performed based on the combined interpretation of ¹H NMR, ¹³C NMR, and HSQC spectra. A complete list of the chemical shift values (δ), signal multiplicities, and coupling constants has now been included in the manuscript. In addition, the full raw NMR spectra (¹H NMR, ¹³C NMR, and HSQC) have been provided as Supporting Information, clearly labeled with the compound name and structure to facilitate verification of the spectral assignments.

**Changes made:**

Methods section – Compound characterization Supporting Information – NMR spectra.

**Comment 4**

Provide information regarding field collection permits.

**Response:**
Thank you for this important comment. We have added a statement in the *Plant material* subsection of the Methods to clarify the authorization for plant collection. Permission for plant collection was obtained from the Ethiopian Wildlife Conservation Authority (EWCA), the authority responsible for managing Awash National Park, and the collection was conducted in accordance with national regulations. The manuscript now clearly indicates that the study did not involve endangered or protected plant species.

**Changes made:**

Methods section – Plant collection and identification.

**Comment 5**

Update submission using the PLOS LaTeX template.

**Response:**
Thank you for this guidance. We have revised the manuscript to comply with the PLOS ONE LaTeX template requirements. The manuscript has been reformatted using the official PLOS LaTeX template, and all sections—including the title page, abstract, main text, references, figures, tables, and Supporting Information—have been adjusted to meet the journal’s formatting and file-naming guidelines. These revisions were made to ensure consistency with PLOS ONE submission standards.

**Comment 6–9**

Funding statement clarification and role of funders.

**Response:**
We thank the Editor for pointing this out. The funding information has been corrected to ensure consistency. Funding information has been removed from the Acknowledgments section and included only in the Funding Statement. The role of the funder has also been clarified.

**Revised statement:**

“The study was supported by the Office of the Vice President for Research and Innovation of Addis Ababa University (Grant number: TR/014/2021). The funders had no role in study design, data collection and analysis, decision to publish, or preparation of the manuscript.”

**Changes made:**

Funding Statement section.

**Comment 10**

Ensure abstract consistency between submission form and manuscript.

**Response:**
We have carefully reviewed the abstract in both the manuscript and the online submission form. The text has been updated to ensure complete consistency between the two, including all numerical data, compound names, and statistical details. No discrepancies remain.

**Comment 11**

Move ethics statement to Methods section only.

**Response:**
The ethics statement has been moved exclusively to the Methods section and removed from other sections.

**Comment 12**

Include captions for Supporting Information.

**Response:**
Captions for all Supporting Information files have now been included at the end of the manuscript.

**Comment 13**

Evaluate recommended citations.

**Response:**
All recommended references were carefully evaluated and incorporated where scientifically relevant.

**Additional Editor Major Concerns**

**Ethical and Scientific Justification for Animal Use**

**Response:**
We appreciate the Editor’s concern regarding the justification for animal experiments. The Introduction has been expanded to provide stronger literature-based evidence supporting the ethnomedicinal use of *Grewia schweinfurthii* for pain and inflammatory conditions, as well as prior studies demonstrating pharmacological potential in related species. This additional context provides a clearer scientific rationale for conducting the *in vivo* evaluation.

**Changes made:**

Introduction section expanded.

**Insufficient Chemical Characterization**

**Response:**
We thank the editor for this important observation. The Methods section has been expanded to provide full details of the chemical characterization of the isolated compound AL-03 (4-(2''-(4'-isopropylphenyl) propan-2''-yl)-2,3-dihydrofuran). This now includes: the NMR instrument model (Bruker Avance DMX 400), magnetic field strength (400 MHz for ¹H and 100 MHz for ¹³C), nuclei measured, solvent system (CDCl₃), reference standard (TMS), and all experimental acquisition conditions. Chemical shift data, signal multiplicities, coupling constants, and structural assignment using HSQC are now explicitly reported. Purity was confirmed by reproducible TLC analysis in two solvent systems, and the full NMR spectra (¹H, ¹³C, HSQC) are provided as Supporting Information (S4–S6 Figs). These revisions address concerns regarding structural elucidation, purity assessment, and transparency of compound characterization.

**Unusually High Anti-inflammatory Efficacy**

**Response:**
We thank the Editor for highlighting this issue. The calculation method used for paw edema inhibition has been clarified, and the raw paw volume changes and corresponding statistical analysis have been carefully verified and reported.

**Changes made:**

Results section – Anti-inflammatory activity.

**Mechanistic Overinterpretation**

**Response:**
We thank the Editor for this important observation. We agree that mechanistic interpretations should be presented cautiously. Accordingly, statements in the manuscript suggesting COX inhibition, prostaglandin suppression, or modulation of inflammatory mediators have been revised. We now describe these as plausible mechanisms based on existing literature, rather than direct findings from our study. This clarification has been incorporated under the subsection “Cohesive Integration of Peripheral, Central, and Anti-inflammatory Effects” in the Discussion, emphasizing that while the extract and isolated compound demonstrated significant analgesic and anti-inflammatory effects *in vivo*, the precise molecular pathways remain to be experimentally confirmed.

**Changes made:**

Discussion section revised.

**Data Availability**

**Response:**
All underlying individual-level data used in the analyses have now been provided as Supporting Information material, in compliance with PLOS ONE data availability requirements.

**Reviewer #1**

**Comment: Include numeric results in the Abstract.**

**Response:**
Key numerical findings (percentage inhibition and latency values) have been added to the Abstract.

**Comment: Clarify research gap and strengthen rationale.**

**Response:**
The Introduction has been revised to clearly identify the research gap regarding the pharmacological evaluation of *Grewia schweinfurthii*.

**Comment: Provide voucher specimen number.**

**Response:**
The voucher specimen number has now been included in the Methods section.

**Comment: Report extraction yield and storage conditions.**

**Response:**
Extraction yield (%) and storage conditions have now been added.

**Comment: Remove redundancy in phytochemical screening.**

**Response:**
The duplicated mention of flavonoids has been corrected.

**Comment: Clarify OECD guideline for toxicity.**

**Response:**
The OECD guideline used for acute toxicity testing has now been clearly specified.

**Comment: Provide baseline latency and cutoff time for hot plate test.**

**Response:**
Baseline latency and maximum cutoff time have now been added.

**Comment: Improve carrageenan assay description.**

**Response:**
The injection site, phases of inflammation, and measurement details have been clarified.

**Comment: Report yield and purity of isolated compound.**

**Response:**
The yield and characterization data for compound AL-03 have been included, along with purity assessment information.

**Comment: Avoid unsupported mechanistic claims.**

**Response:**
Mechanistic explanations have been revised to avoid overinterpretation and are now presented cautiously with literature support.

**Comment: Include time-course figures.**

**Response:**
Time-course graphs for both **hot plate latency** and **paw edema inhibition** have been included with appropriate statistical annotations.

**Comment: Add study limitations and future recommendations.**

**Response:**
A paragraph describing study limitations and recommendations for future research has been added to the Conclusion section.

**Reviewer #2**

**Comment: Title may be misleading.**

**Response:**
Thank you for this important observation. While the isolated compound was chemically characterized, it was also evaluated for anti-inflammatory activity in the carrageenan-induced paw edema model. To accurately reflect both the pharmacological evaluation of the extract and the experimental assessment of the isolated compound, the title has been revised.

**Comment: Improve abstract (objective, models, dosages, statistics).**

**Response:**
The Abstract has been revised to include the study objective, experimental models, treatment doses, OECD guideline, statistical analysis method, and key numeric results.

**Comment: Improve clarity and focus in Introduction.**

**Response:**
The Introduction has been edited to improve clarity, reduce unnecessary botanical descriptions, and clearly define the research gap and study objectives.

**Comment: Improve methodological transparency.**

**Response:**
Additional methodological details have been added including animal species, randomization procedures, route of administration, ethical approval, and measurement techniques.

**Comment: Clarify LD50 interpretation.**

**Response:**
The toxicity experiment has been clarified as a limit test for preliminary acute toxicity assessment rather than a formal LD50 determination.

**Comment: Clarify TLC methodology.**

**Response:**
Additional explanation of solvent systems and TLC interpretation has been included.

**Comment: Improve statistical reporting.**

**Response:**
Statistical methods have been clarified in the Statistical Analysis section. Statistical significance levels are reported in the Results and figures.

**Comment: Improve discussion and comparison with previous studies.**

**Response:**
The Discussion has been revised to clearly separate results from interpretation, reduce speculation, and strengthen comparison with previously published studies.

**Comment: Revise conclusion.**

**Response:**
The Conclusion has been rewritten to clearly state that findings are derived from animal models, acknowledge study limitations, and recommend future studies including mechanistic investigations and further pharmacological validation.

**Final Statement**

We sincerely thank the Editor and the Reviewers for their constructive comments. We believe that the revisions made in response to these comments have significantly improved the clarity, rigor, and transparency of the manuscript, and we hope that the revised version will now be suitable for publication in *PLOS ONE*.
