# Supplementary material for: Evaluation of analgesic and anti-inflammatory activities of the root extract of Grewia schweinfurthii Burret and its major chemical constituents
Source: PLoS One. 2026 Jul 22;21(7):e0353400. doi: 10.1371/journal.pone.0353400 (PMC13483540; doi:10.1371/journal.pone.0353400)
Supplement: Supplementary file 4 [file pone.0353400.s004.docx]

**Peer Review**

1. The scientific rationale of the study remains relatively generic and insufficiently developed: considering that several studies on Grewia species, including analgesic and anti-inflammatory activities, are already available, what is the actual conceptual advance of this work beyond incremental validation? Could the authors more critically clarify the real scientific gap addressed, particularly regarding pharmacological or phytochemical novelty?
2. The experimental transition from a crude extract (80% methanolic) to the investigation of a single isolated compound lacks clear conceptual integration: what was the rationale guiding this approach, and is there evidence that compound AL-03 is the primary contributor to the observed biological activity of the extract?
3. The phytochemical characterization is largely qualitative and limited: how do the authors justify the absence of quantitative analysis of major metabolites (e.g., total phenolics, flavonoids), which are essential for pharmacological correlation?
4. Although supported by NMR data, the structural elucidation of the isolated compound lacks comprehensive analytical validation: why were complementary techniques such as LC-MS/MS or HRMS not employed to confirm the structure more robustly?
5. The reported dose-response relationship raises important concerns: how do the authors explain the apparent equivalence between the crude extract (400 mg/kg) and the standard drug (indomethacin), given the lack of extract standardization? Is there a risk of overinterpretation of efficacy?
6. The experimental design does not clearly indicate whether a sample size calculation (power analysis) was performed: how do the authors ensure that n=6 per group is sufficient to detect biologically meaningful differences?
7. Randomization and blinding procedures are not clearly described: were the investigators blinded to treatment allocation during behavioral assessments such as the hot plate and writhing tests?
8. The use of animals of both sexes without stratification or sex-based analysis may introduce variability: did the authors consider sex-related differences in inflammatory and nociceptive responses?
9. Dose selection for the extract appears to be based on a simplified rationale derived from acute toxicity data: why was a broader dose-response curve or ED50 determination not performed?
10. In the carrageenan-induced edema model, mechanistic interpretations are proposed without direct biochemical measurements: how do the authors support claims regarding involvement of specific inflammatory mediators (e.g., COX, prostaglandins, NO)?
11. The results indicate very high efficacy (e.g., up to 93% inhibition), yet lack critical discussion regarding variability and biological relevance: how do the authors contextualize these findings within the existing literature without overstating efficacy?
12. The exclusive use of one-way ANOVA followed by Tukey’s post hoc test may not be appropriate for time-course data (e.g., hot plate and carrageenan models): did the authors consider statistical approaches suitable for repeated measures?
13. Statistical significance (p < 0.05) is frequently interpreted as biological relevance: could the authors clearly distinguish between statistical significance and effect size?
14. Confidence intervals are not reported: why were they not included, given their importance for data interpretation?
15. Mechanistic conclusions (e.g., inhibition of prostaglandin synthesis or COX pathways) are largely speculative: how are these claims justified in the absence of direct mechanistic assays?
16. Given the biphasic nature of carrageenan-induced inflammation, could the authors more critically discuss which phase is predominantly affected by the extract and the implications of this finding?
17. The manuscript suggests potential therapeutic applicability: how do the authors justify this translational inference without pharmacokinetic, bioavailability, or subchronic toxicity data?
18. Some conceptual inconsistencies are present, such as statements implying that acute inflammation is minimally beneficial or may worsen infections, which contradicts established immunological principles: could the authors revise these interpretations?
19. Although improved, methodological descriptions still lack full standardization in certain sections (e.g., environmental conditions, preparation procedures): how do the authors ensure reproducibility?
20. The Results and Discussion sections remain largely descriptive rather than analytically integrated: why did the authors not adopt a more critical, comparative discussion with existing studies?
21. The manuscript does not explicitly address its limitations: what are the main methodological and interpretative limitations acknowledged by the authors?
