# Supplementary material for: Evaluation of analgesic and anti-inflammatory activities of the root extract of Grewia schweinfurthii Burret and its major chemical constituents
Source: PLoS One. 2026 Jul 22;21(7):e0353400. doi: 10.1371/journal.pone.0353400 (PMC13483540; doi:10.1371/journal.pone.0353400)
Supplement: Supplementary file 7 [file pone.0353400.s007.docx]

**Response to Reviewer**

**Manuscript Title:** Evaluation of analgesic and anti-inflammatory activities of the root extract of *Grewia schweinfurthii* Burret and its major chemical constituents

**Manuscript ID:** PONE-D-26-01189

**Journal:** PLOS ONE

**Date:** Jun 08, 2026

We sincerely thank the Academic Editor and the Peer Reviewer for their time and constructive appraisal of our manuscript. The comments have substantially improved the scientific rigor and transparency of this work. We provide a point-by-point response to each comment below; all changes are highlighted in the accompanying tracked-changes manuscript.

──────────────────────────────────────────────────────────────────────────────────────────

**PEER REVIEWER**

We sincerely thank the Peer Reviewer for the comprehensive and detailed critique. The 21 comments have collectively prompted significant improvements in scientific rigor and transparency. Where experimental revision is not feasible at this stage, we provide transparent explanations and have explicitly acknowledged remaining limitations in a dedicated new section.

**Comment 1:** *The scientific rationale of the study remains relatively generic and insufficiently developed: considering that several studies on Grewia species are already available, what is the actual conceptual advance of this work beyond incremental validation?*

**Authors’ Response:** We appreciate this important comment and have revised the final paragraph of the Introduction to better articulate the conceptual and methodological significance of the study. Specifically, we now clarify that: (i) no prior study has evaluated the analgesic and anti-inflammatory activities of *G. schweinfurthii* root extract specifically; (ii) the isolated compound AL-03 (a dihydrofuran derivative) has not been previously reported from this species; and (iii) the simultaneous dual evaluation of a crude extract and a structurally characterized compound using complementary peripheral, central, and inflammatory models is a methodological advance over prior studies on related *Grewia* species, which predominantly examined extracts alone. The revised Introduction now positions the study as providing the first systematic evidence for this under-studied species, bridging the gap between traditional use and scientific validation.

**Comment 2:** *The experimental transition from a crude extract (80% methanolic) to the investigation of a single isolated compound lacks clear conceptual integration: what was the rationale guiding this approach, and is there evidence that compound AL-03 is the primary contributor to the observed biological activity?*

**Authors’ Response:** We appreciate this important observation and have revised both the Methods and Discussion to clarify the rationale for transitioning from crude extract evaluation to compound isolation. In the Isolation section, we now explain that AL-03 was selected because it was obtained as a major constituent from the most biologically active fractions during chromatographic separation. The extract-to-compound approach was intended to provide preliminary insight into the phytochemical components potentially associated with the observed pharmacological activities and to identify structurally characterized constituents for future mechanistic investigation.

We acknowledge, however, that the present study was not designed to establish AL-03 as the sole or primary contributor to the overall activity of the crude extract. Accordingly, the Discussion now states: “The contribution of AL-03 to the extract’s overall activity cannot be determined from the current data alone; the extract activity most likely reflects the combined pharmacological effects of multiple constituents, and AL-03 represents a single characterized component warranting further investigation.” We also note that synergistic interactions among phytochemicals are common in medicinal plant extracts and may substantially contribute to the observed biological effects.

**Comment 3:** *The phytochemical characterization is largely qualitative and limited: how do the authors justify the absence of quantitative analysis of major metabolites (e.g., total phenolics, flavonoids)?*

**Authors’ Response:** We acknowledge this as an important limitation of the study. Quantitative determination of major metabolite classes, including total phenolics and flavonoids, was not performed in the present investigation. The primary objective of this work was to conduct an initial pharmacological evaluation of the analgesic and anti-inflammatory activities of *Grewia schweinfurthii*, together with preliminary phytochemical characterization and compound isolation. Accordingly, the study emphasized bioactivity assessment and structural identification of selected constituents rather than comprehensive quantitative metabolite profiling.

We have now explicitly acknowledged this limitation in the newly added Limitations section and identified quantitative phytochemical and metabolomics-based analyses as important priorities for future work. We agree that such analyses would strengthen understanding of the relationship between phytochemical composition and observed biological activity.

**Comment 4:** *Although supported by NMR data, the structural elucidation of the isolated compound lacks comprehensive analytical validation: why were complementary techniques such as LC-MS/MS or HRMS not employed?*

**Authors’ Response:** We appreciate this important comment. The structural assignment of AL-03 was based on the combined interpretation of ^1H NMR, ^13C NMR, and HSQC spectroscopic data, which provided internally consistent evidence supporting the proposed structure. We agree, however, that complementary analytical techniques such as HRMS or LC-MS/MS would provide additional confirmation of the molecular formula and strengthen structural validation.

Due to the scope and analytical resources available for the present study, these complementary analyses were not performed. We have therefore explicitly acknowledged this as a limitation in the revised manuscript and added the following statement: “Structural assignment based solely on NMR data represents a limitation of the present study; future investigations should incorporate HRMS or LC-MS/MS analyses to confirm the molecular formula and provide independent structural validation of AL-03.” We nevertheless note that the NMR data obtained were sufficiently coherent for preliminary structural characterization of the isolated compound.

**Comment 5:** *The reported dose-response relationship raises important concerns: how do the authors explain the apparent equivalence between the crude extract (400 mg/kg) and the standard drug (indomethacin), given the lack of extract standardization? Is there a risk of overinterpretation of efficacy?*

**Authors’ Response:** We agree and have revised the Discussion accordingly. A new passage clarifies that the 93% inhibition at 400 mg/kg (extract) versus 95% for indomethacin at 10 mg/kg represents a 40-fold dose difference. Since the extract was not standardized, this does not constitute potency equivalence. The revised text states: "This dose difference of 40-fold, combined with the absence of extract standardization, precludes direct potency comparison with indomethacin. The results are reported in the context of efficacy demonstration rather than clinical equivalence, and any inference of comparable anti-inflammatory potency would be an overinterpretation of the current data."

**Comment 6:** *The experimental design does not clearly indicate whether a sample size calculation (power analysis) was performed: how do the authors ensure that n=6 per group is sufficient?*

**Authors’ Response:** We appreciate the reviewer’s important comment. A formal a priori power analysis was not conducted prior to the study. The sample size of n = 6 animals per group was selected based on established practice in comparable in vivo analgesic and anti-inflammatory studies, together with ethical considerations aimed at minimizing animal use in accordance with standard preclinical research principles.

To improve transparency, we have added the following statement to the Methods section: “Formal a priori power analysis was not performed; sample size was determined based on previously published studies using similar experimental models. Therefore, the possibility that smaller effect sizes may not have been detected cannot be excluded, representing a limitation of the study.”

Although statistically significant differences were observed for the primary outcomes assessed, we acknowledge that the absence of prospective power analysis limits the statistical rigor of the study. Future investigations will incorporate formal sample size estimation to strengthen experimental design and statistical robustness.

**Comment 7:** *Randomization and blinding procedures are not clearly described: were the investigators blinded to treatment allocation during behavioral assessments?*

**Authors’ Response:** We have clarified in the Methods that animals were allocated to groups by random number assignment. Blinding was applied to all behavioral observations: writhing counts, hot plate latency measurements, and paw volume measurements were all conducted by an investigator unaware of group assignments. This has been explicitly stated in the revised Methods section.

**Comment 8:** *The use of animals of both sexes without stratification or sex-based analysis may introduce variability: did the authors consider sex-related differences in inflammatory and nociceptive responses?*

**Authors’ Response:** We acknowledge the reviewer’s concern regarding potential sex-related variability. Animals of both sexes were included in the study, and sex was distributed as evenly as possible across experimental groups; however, no formal sex-stratified randomization or sex-based subgroup analysis was performed. We recognize that sex-related differences in nociceptive thresholds and inflammatory responses have been reported in the literature and may contribute to variability in experimental outcomes. This limitation has now been explicitly stated in both the Methods and Limitations sections of the revised manuscript. Future studies should incorporate appropriately powered sex-stratified designs to better evaluate potential sex-specific effects.

**Comment 9:** *Dose selection for the extract appears to be based on a simplified rationale derived from acute toxicity data: why was a broader dose-response curve or ED50 determination not performed?*

**Authors’ Response:** The extract doses of 100, 200, and 400 mg/kg were selected based on fractions (1/20th, 1/10th, and 1/5th, respectively) of the 2000 mg/kg limit dose identified during the acute oral toxicity study. This approach is commonly used in exploratory in vivo pharmacological investigations of medicinal plant extracts to identify biologically active dose ranges while maintaining an appropriate safety margin. Given that this study represents the first pharmacological evaluation of *G. schweinfurthii*, the selected doses were intended to provide an initial assessment of analgesic and anti-inflammatory activity rather than a full pharmacodynamic characterization.

We acknowledge that a broader dose-response evaluation and formal ED50 determination would provide a more precise characterization of potency and efficacy. These limitations have been acknowledged in the revised manuscript and will be addressed in future mechanistic and pharmacodynamic studies.

**Comment 10:** *In the carrageenan-induced edema model, mechanistic interpretations are proposed without direct biochemical measurements: how do the authors support claims regarding involvement of specific inflammatory mediators?*

**Authors’ Response:** We fully acknowledge that no direct biochemical measurements were performed. All mechanistic statements in the Discussion have been revised to include clear qualifiers such as "is consistent with," "may plausibly involve," or "suggests, though does not confirm." The Limitations section now explicitly states: "No direct biochemical mechanistic assays were performed; all mechanistic interpretations are inferential and require confirmation by targeted in vitro or ex vivo experiments."

**Comment 11:** *The results indicate very high efficacy (e.g., up to 93% inhibition), yet lack critical discussion regarding variability and biological relevance.*

**Authors’ Response:** We appreciate the reviewer’s important observation regarding the high percentage inhibition values observed in the carrageenan-induced paw edema model. We have expanded the Discussion to clarify that: (i) the carrageenan model is a sensitive model with reliably high inhibition values in the literature; (ii) the 93% inhibition at 400 mg/kg should be interpreted relative to the 40-fold dose difference versus indomethacin; and (iii) these are preliminary proof-of-concept findings. Definitive efficacy claims require dose-response modeling, ED50 determination, and mechanistic validation.

**Comment 12:** *The exclusive use of one-way ANOVA followed by Tukey's post hoc test may not be appropriate for time-course data: did the authors consider statistical approaches suitable for repeated measures?*

**Authors’ Response:** We appreciate the reviewer’s important statistical observation. Because the time-course measurements were obtained repeatedly from the same animals, repeated-measures ANOVA or linear mixed-effects modeling would indeed represent a more statistically appropriate analytical approach, as these methods account for within-subject correlation across time points. In the present study, one-way ANOVA followed by Tukey’s post hoc test was applied independently at each time point, which does not fully account for the repeated-measures structure of the data.

To address this concern, we have explicitly acknowledged this limitation in both the Statistical Analysis and Limitations sections of the revised manuscript. The revised text now states: “One-way ANOVA applied independently at each time point does not account for the within-subject correlation inherent in repeated-measures designs; therefore, repeated-measures ANOVA or linear mixed-effects models would represent more appropriate statistical approaches for analysis of the time-course data.”

**Comment 13:** *Statistical significance (p < 0.05) is frequently interpreted as biological relevance: could the authors clearly distinguish between statistical significance and effect size?*

**Authors’ Response:** We agree. We have added a clarifying statement to the Statistical Analysis section noting that a p-value represents the probability of obtaining results as extreme as observed *assuming the null hypothesis is true*, and should not be interpreted as a measure of effect magnitude or biological importance. We acknowledge that effect sizes (e.g., Cohen's *d* or partial eta-squared) and 95% confidence intervals were not reported in the current manuscript, which is an additional limitation. Where feasible, these will be incorporated in future work, and we have noted this explicitly in the revised Limitations section.

**Comment 14:** *Confidence intervals are not reported: why were they not included, given their importance for data interpretation?*

**Authors’ Response:** The reviewer raises a valid point. Mean ± SEM was reported as the primary summary statistic, and 95% confidence intervals were not included in the original manuscript. Since CIs can be derived from the existing mean, SEM, and group size (n = 6), we have calculated and added 95% CIs to the relevant tables in the revised manuscript, providing a more complete picture of estimate precision. We acknowledge that CI reporting is increasingly expected for transparent and reproducible pharmacological research, and the revised manuscript now conforms to this standard.

**Comment 15:** *Mechanistic conclusions (e.g., inhibition of prostaglandin synthesis or COX pathways) are largely speculative: how are these claims justified in the absence of direct mechanistic assays?*

**Authors’ Response:** We fully concur and have revised the manuscript throughout to clearly distinguish observations from inferences. All mechanistic statements are now explicitly framed as hypotheses or plausible inferences. Phrases such as "these findings demonstrate COX inhibition" have been replaced with "these findings are consistent with, but do not confirm, inhibition of prostaglandin synthesis pathways." The Limitations section explicitly states that direct mechanistic confirmation requires targeted biochemical assays.

**Comment 16:** *Given the biphasic nature of carrageenan-induced inflammation, could the authors more critically discuss which phase is predominantly affected by the extract?*

**Authors’ Response:** We thank the reviewer for this insightful comment. A new paragraph has been added to the Discussion to more critically interpret the biphasic nature of carrageenan-induced inflammation. As shown in Table 4, the extract exhibited comparatively higher inhibition during the later phase (3–5 h; 84–93% at 400 mg/kg) than in the early phase (1–2 h; 71% at 400 mg/kg). This pattern suggests that the anti-inflammatory effect may be more pronounced during the late phase, which is generally associated with prostaglandin-mediated responses. While this observation is consistent with a possible involvement of pathways linked to prostaglandin synthesis, such as COX-related mechanisms, it does not provide direct evidence of such activity. Similarly, the lower inhibition observed in the early phase suggests that mediator systems such as histamine and serotonin may be less affected; however, this remains speculative in the absence of targeted biochemical assays.

**Comment 17:** *The manuscript suggests potential therapeutic applicability: how do the authors justify this translational inference without pharmacokinetic, bioavailability, or sub-chronic toxicity data?*

**Authors’ Response:** All references to therapeutic applicability have been revised to explicitly note that current findings are preliminary and animal-based. The Conclusion now states: "These findings provide a scientific basis for further investigation but do not constitute evidence of therapeutic efficacy or safety in humans. Pharmacokinetic profiling, bioavailability assessment, and sub-chronic toxicity studies are prerequisites before any translational inference can be made."

**Comment 18:** *Some conceptual inconsistencies are present, such as statements implying that acute inflammation is minimally beneficial or may worsen infections, which contradicts established immunological principles.*

**Authors’ Response:** We thank the reviewer for identifying this inaccuracy. The statement has been revised to correctly acknowledge the protective role of acute inflammation while accurately characterizing the consequences of dysregulation. The revised text reads:

*"While acute inflammation is a necessary and beneficial component of host defense, a dysregulated or excessively prolonged acute inflammatory response can lead to collateral tissue injury and contribute to worse disease outcomes."*

**Comment 19:** *Although improved, methodological descriptions still lack full standardization in certain sections (e.g., environmental conditions, preparation procedures): how do the authors ensure reproducibility?*

**Authors’ Response:** We have reviewed the Methods and added further detail. Environmental conditions (12-hour light-dark cycle, 22 ± 2°C, 50–60% humidity, free access to food and water) are described in the Experimental Animals section. Extraction procedure, yield (12.6% w/w), drying conditions, and storage (4°C, airtight amber containers) are described in the Plant Material Preparation section. All procedural details sufficient for replication are now included.

**Comment 20:** *The Results and Discussion sections remain largely descriptive rather than analytically integrated: why did the authors not adopt a more critical, comparative discussion with existing studies?*

**Authors’ Response:** We have substantially revised the Results and Discussion to improve analytical integration: (i) a comparative discussion contextualizing extract efficacy relative to other Grewia species and Ethiopian medicinal plant studies was added; (ii) effect sizes relative to reference drugs are discussed more critically; (iii) phytochemical data are more explicitly integrated with pharmacological findings linking detected classes to the observed phase-specific anti-inflammatory profile; and (iv) the Cohesive Integration subsection synthesizes findings across all models.

**Comment 21:** *The manuscript does not explicitly address its limitations: what are the main methodological and interpretative limitations acknowledged by the authors?*

**Authors’ Response:** A dedicated Limitations section has been added immediately before the Declarations section. It enumerates nine key limitations: (1) use of acute models only; (2) qualitative phytochemical characterization; (3) absence of HRMS structural confirmation for AL-03; (4) lack of pharmacokinetic/toxicokinetic data; (5) no direct mechanistic biochemical assays; (6) sample size based on convention rather than power analysis; (7) absence of sex stratification; (8) one-way ANOVA applied to time-course data; and (9) absence of extract standardization.

──────────────────────────────────────────────────────────────────────────────────────────

We believe these revisions comprehensively address all concerns raised and substantially strengthen the scientific rigor and transparency of the manuscript. We remain available to provide any additional clarification.

Sincerely,

**Abdi Leta Gemechu** (Corresponding Author)

Ambo University, College of Medicine and Health Science, Department of Pharmacy

Email: abdi.leta@ambou.edu.et

On behalf of all co-authors: Mirutse Giday, Solomon Tadesse, Ariaya Hymete
