# Supplementary material for: Evaluation of analgesic and anti-inflammatory activities of the root extract of Grewia schweinfurthii Burret and its major chemical constituents
Source: PLoS One. 2026 Jul 22;21(7):e0353400. doi: 10.1371/journal.pone.0353400 (PMC13483540; doi:10.1371/journal.pone.0353400)
Supplement: Peer review history

# Peer review: Decision Letter 0

**DOI:** 10.1371/journal.pone.0353400.r001

**Authors:** Eshetie Melese Birru

26 Feb 2026

PONE-D-26-01189

Evaluation of analgesic and anti-inflammatory activities of the root extract of Grewia schweinfurthii Burret and its major chemical constituents

PLOS One

Dear Dr. Gemechu,

Thank you for submitting your manuscript to PLOS ONE. After careful consideration, we feel that it has merit but does not fully meet PLOS ONE’s publication criteria as it currently stands. Therefore, we invite you to submit a revised version of the manuscript that addresses the points raised during the review process.

Please submit your revised manuscript by Apr 12 2026 11:59PM. If you will need more time than this to complete your revisions, please reply to this message or contact the journal office at plosone@plos.org. When you're ready to submit your revision, log on to https://www.editorialmanager.com/pone/ and select the 'Submissions Needing Revision' folder to locate your manuscript file.

Please include the following items when submitting your revised manuscript:

- A letter that responds to each point raised by the academic editor and reviewer(s). You should upload this letter as a separate file labeled 'Response to Reviewers'.
- A marked-up copy of your manuscript that highlights changes made to the original version. You should upload this as a separate file labeled 'Revised Manuscript with Track Changes'.
- An unmarked version of your revised paper without tracked changes. You should upload this as a separate file labeled 'Manuscript'.

If you would like to make changes to your financial disclosure, please include your updated statement in your cover letter. Guidelines for resubmitting your figure files are available below the reviewer comments at the end of this letter.

If applicable, we recommend that you deposit your laboratory protocols in protocols.io to enhance the reproducibility of your results. Protocols.io assigns your protocol its own identifier (DOI) so that it can be cited independently in the future. For instructions see: https://journals.plos.org/plosone/s/submission-guidelines#loc-laboratory-protocols. Additionally, PLOS ONE offers an option for publishing peer-reviewed Lab Protocol articles, which describe protocols hosted on protocols.io. Read more information on sharing protocols at https://plos.org/protocols?utm_medium=editorial-email&utm_source=authorletters&utm_campaign=protocols.

We look forward to receiving your revised manuscript.

Kind regards,

Eshetie Melese Birru, PhD

Academic Editor

PLOS One

Journal Requirements:

When submitting your revision, we need you to address these additional requirements.

1. Please ensure that your manuscript meets PLOS ONE's style requirements, including those for file naming. The PLOS ONE style templates can be found at

https://journals.plos.org/plosone/s/file?id=wjVg/PLOSOne_formatting_sample_main_body.pdf and

https://journals.plos.org/plosone/s/file?id=ba62/PLOSOne_formatting_sample_title_authors_affiliations.pdf

2. To comply with PLOS One submissions requirements, in your Methods section, please provide additional information regarding the experiments involving animals and ensure you have included details on (1) methods of sacrifice, (2) methods of anesthesia and/or analgesia, and (3) efforts to alleviate suffering.

3. We note that this submission includes NMR spectroscopy data. We would recommend that you include the following information in your methods section or as Supporting Information files:

a) The make/source of the NMR instrument used in your study, as well as the magnetic field strength. For each individual experiment, please also list: the nucleus being measured; the sample concentration; the solvent in which the sample is dissolved and if solvent signal suppression was used; the reference standard and the temperature.

b) A list of the chemical shifts for all compounds characterised by NMR spectroscopy, specifying, where relevant: the chemical shift (δ), the multiplicity and the coupling constants (in Hz), for the appropriate nuclei used for assignment.

c)The full integrated NMR spectrum, clearly labelled with the compound name and chemical structure.

We also strongly encourage authors to provide primary NMR data files, in particular for new compounds which have not been characterised in the existing literature. Authors should provide the acquisition data, FID files and processing parameters for each experiment, clearly labelled with the compound name and identifier, as well as a structure file for each provided dataset. See our list of recommended repositories here: https://journals.plos.org/plosone/s/recommended-repositories

4. In your Methods section, please provide additional information regarding the permits you obtained for the work. Please ensure you have included the full name of the authority that approved the field site access and, if no permits were required, a brief statement explaining why.

5. Please update your submission to use the PLOS LaTeX template. The template and more information on our requirements for LaTeX submissions can be found at http://journals.plos.org/plosone/s/latex.

6. Thank you for stating the following in the Acknowledgments Section of your manuscript:

“The study was sponsored by the Office of the Vice President for Research and Innovation of the Addis Ababa University (Grant number: TR/014/2021).”

We note that you have provided funding information that is not currently declared in your Funding Statement. However, funding information should not appear in the Acknowledgments section or other areas of your manuscript. We will only publish funding information present in the Funding Statement section of the online submission form.

Please remove any funding-related text from the manuscript and let us know how you would like to update your Funding Statement. Currently, your Funding Statement reads as follows:

“The study was sponsored by the Office of the Vice President for Research and Innovation of Addis Ababa University (Grant number: TR/014/2021).”

Please include your amended statements within your cover letter; we will change the online submission form on your behalf.

7. We note that the grant information you provided in the ‘Funding Information’ and ‘Financial Disclosure’ sections do not match.

When you resubmit, please ensure that you provide the correct grant numbers for the awards you received for your study in the ‘Funding Information’ section.

8. Thank you for stating the following financial disclosure:

“The study was sponsored by the Office of the Vice President for Research and Innovation of Addis Ababa University (Grant number: TR/014/2021).”

Please state what role the funders took in the study.  If the funders had no role, please state: "The funders had no role in study design, data collection and analysis, decision to publish, or preparation of the manuscript."

If this statement is not correct you must amend it as needed.

Please include this amended Role of Funder statement in your cover letter; we will change the online submission form on your behalf.

9. Thank you for stating the following financial disclosure:

“The study was sponsored by the Office of the Vice President for Research and Innovation of Addis Ababa University (Grant number: TR/014/2021).”

We note that one or more of the authors is affiliated with the funding organization, indicating the funder may have had some role in the design, data collection, analysis or preparation of your manuscript for publication; in other words, the funder played an indirect role through the participation of the co-authors. If the funding organization did not play a role in the study design, data collection and analysis, decision to publish, or preparation of the manuscript and only provided financial support in the form of authors' salaries and/or research materials, please do the following:

1. Review your statements relating to the author contributions, and ensure you have specifically and accurately indicated the role(s) that these authors had in your study. These amendments should be made in the online form.

2. Confirm in your cover letter that you agree with the following statement, and we will change the online submission form on your behalf:

“The funder provided support in the form of salaries for authors [insert relevant initials], but did not have any additional role in the study design, data collection and analysis, decision to publish, or preparation of the manuscript. The specific roles of these authors are articulated in the ‘author contributions’ section.

10. Please amend either the abstract on the online submission form (via Edit Submission) or the abstract in the manuscript so that they are identical.

11. Your ethics statement should only appear in the Methods section of your manuscript. If your ethics statement is written in any section besides the Methods, please move it to the Methods section and delete it from any other section. Please ensure that your ethics statement is included in your manuscript, as the ethics statement entered into the online submission form will not be published alongside your manuscript.

12. Please include captions for your Supporting Information files at the end of your manuscript, and update any in-text citations to match accordingly. Please see our Supporting Information guidelines for more information: http://journals.plos.org/plosone/s/supporting-information.

13. If the reviewer comments include a recommendation to cite specific previously published works, please review and evaluate these publications to determine whether they are relevant and should be cited. There is no requirement to cite these works unless the editor has indicated otherwise.

Additional Editor Comments:

Thank you for submitting your manuscript entitled “Evaluation of analgesic and anti-inflammatory activities of the root extract of Grewia schweinfurthii Burret and its major chemical constituents.”While the study uses established in vivo pharmacological models, several substantive concerns must be addressed before further consideration.

Major Concerns

Ethical and Scientific Justification for Animal Use

The manuscript does not clearly demonstrate sufficient prior in vitro, in vivo, or species-specific literature supporting analgesic or anti-inflammatory activity of G. schweinfurthii. In the absence of robust preliminary evidence, direct multi-dose in vivo testing of a crude extract raises concerns under the animal ethics framework. The authors must provide stronger literature-based or experimental justification for progressing to animal experimentation.

Insufficient Chemical Characterization

The structural elucidation of the isolated compound lacks HRMS confirmation, detailed 2D NMR correlation discussion, and purity assessment prior to biological testing. Stronger analytical validation is required.

Unusually High Anti-inflammatory Efficacy

The reported 93% inhibition at 400 mg/kg, comparable to indomethacin, is unusually high for a crude extract. The edema calculation method, raw ΔV data, and statistical handling should be clarified to ensure accuracy.

Mechanistic Overinterpretation

Claims regarding COX inhibition, prostaglandin suppression, and ROS modulation are speculative and not supported by mechanistic assays. These interpretations should be reframed cautiously.

Data Availability Compliance

Full underlying individual-level data must be made available in accordance with PLOS ONE policy.

[Note: HTML markup is below. Please do not edit.]

Reviewers' comments:

Reviewer's Responses to Questions

**Comments to the Author**

1. Is the manuscript technically sound, and do the data support the conclusions?

The manuscript must describe a technically sound piece of scientific research with data that supports the conclusions. Experiments must have been conducted rigorously, with appropriate controls, replication, and sample sizes. The conclusions must be drawn appropriately based on the data presented.

Reviewer #1: Yes

Reviewer #2: Yes

**********

2. Has the statistical analysis been performed appropriately and rigorously?

Reviewer #1: Yes

Reviewer #2: Yes

**********

3. Have the authors made all data underlying the findings in their manuscript fully available?

The PLOS Data policy requires authors to make all data underlying the findings described in their manuscript fully available without restriction, with rare exception (please refer to the Data Availability Statement in the manuscript PDF file). The data should be provided as part of the manuscript or its supporting information, or deposited to a public repository. For example, in addition to summary statistics, the data points behind means, medians and variance measures should be available. If there are restrictions on publicly sharing data—e.g. participant privacy or use of data from a third party—those must be specified.

Reviewer #1: Yes

Reviewer #2: Yes

**********

4. Is the manuscript presented in an intelligible fashion and written in standard English?

PLOS ONE does not copyedit accepted manuscripts, so the language in submitted articles must be clear, correct, and unambiguous. Any typographical or grammatical errors should be corrected at revision, so please note any specific errors here.

Reviewer #1: No

Reviewer #2: Yes

**********

5. Review Comments to the Author

Please use the space provided to explain your answers to the questions above. You may also include additional comments for the author, including concerns about dual publication, research ethics, or publication ethics. (Please upload your review as an attachment if it exceeds 20,000 characters)

Reviewer #1: This study investigates the analgesic and anti-inflammatory activities of the 80% methanolic root extract of Grewia schweinfurthii and its isolated compound (AL-03) using established in vivo models. The results demonstrate dose-dependent peripheral and central analgesic effects, as well as significant inhibition of carrageenan-induced paw edema, comparable to standard drugs. Overall, the manuscript addresses an important area of ethnopharmacology, although improvements in data presentation, mechanistic interpretation, and chemical characterization are needed.

Comments

Abstract: Include key numeric results (e.g., % inhibition of writhing or edema, IC₅₀ if applicable).

Introduction: Clearly state the research gap. Introduce the importance of tannins, flavonoids, saponins, etc., in analgesic and anti-inflammatory activity to strengthen the rationale.

Materials and Methods: Include voucher specimen number for reproducibility. Report yield (%) of crude extract. Include storage conditions (temperature, light protection). Phytochemicals screening: Flavonoids mentioned twice — remove redundancy. Acute Toxicity:Clarify which OECD guideline was followed (423 vs 425). Correct inconsistencies in dose explanation. Hot plate assay: state baseline latency, maximum cutoff time clearly. Carrageenan assay: clarify early/late phase interpretation and injection site. Correct TLC plate thickness (200 μm vs 200 μg). Standardize units (mg/kg, µL, mL, °C).

Results and Discussion: Compound isolation: Report yield (mg, %) of AL-03. Confirm purity (TLC/HPLC/melting point). Acute Toxicity:Include quantitative body weight data. Discuss OECD toxicity category. Compound AL-03: Include full characterization data. Report yield (%). Clarify novelty — first isolation from this species? Analgesic Activity (Writhing & Hot Plate): Provide numerical data: mean ± SEM writhes, latency (s), % inhibition, statistical significance. Include time-course figure for hot plate assay. Avoid mechanistic claims without direct evidence. Anti-inflammatory Activity: Provide paw volume data ± SEM for all time points. Include time-course figure with statistical annotation.Mechanistic statements (COX-2, prostaglandin inhibition) should be phrased as plausible or literature-supported. Discuss contribution of flavonoids, terpenoids, and steroids to analgesic/anti-inflammatory effects. Integrate peripheral (writhing) and central (hot plate) analgesic findings with anti-inflammatory results for a cohesive pharmacological narrative. Integrate peripheral (writhing) and central (hot plate) analgesic findings with anti-inflammatory results for a cohesive pharmacological narrative. Add study limitaions and recommendations for the future.

Reviewer #2: Review Report:

Manuscript Number: PONE-D-26-01189

The manuscript Title: Evaluation of analgesic and anti-inflammatory activities of the root extract of Grewia schweinfurthii Burret and its major chemical constituents.

Detailed Comments/Recommendations:

Title: The title could be slightly misleading if the isolated compound was not tested in vivo, because the phrase “and its major chemical constituents” might imply that their pharmacological activity was directly evaluated. If the compound was only characterized chemically and not tested separately, it would be more accurate to say something like:

“…and characterization of its major chemical constituents”

Abstract:

 The objective should be clearly and directly stated in the abstract.

 The animal model used should be mentioned in the abstract.

 The study design along dosages (treatment protocols) are lacking.

 The name of statistical analysis should be briefly mentioned.

 The authors should also mention which OECD guidelines were used.

 Results lack the numerical data. Few numeric data must be mentioned to attract the reader’s interest.

 The conclusion must directly reflect the findings and avoid overstated claims like “can be developed as a drug” etc.

Introduction:

 In 3rd paragraph the sentence “It has a minimally helpful effect, especially when faced with pathogenic difficulties; instead, it makes infections worse” is difficult to understand. Please rephrase it.

 In 5th paragraph please briefly summarize adverse effects in 1–2 sentences instead of listing multiple drug classes and complications.

 In 7th Paragraph, consider shortening botanical and morphological details (height, leaves) unless directly relevant to pharmacological study.

 Please clarify which traditional uses specifically relate to analgesic and anti-inflammatory activity (e.g., joint pain, headache).

 References could be integrated more smoothly; currently the long list of species may distract the reader.

 In last paragraph please clarify which traditional uses specifically relate to analgesic and anti-inflammatory activity (e.g., joint pain, headache).

 There is vague gap statement with mentions that “few species evaluated” but does not clearly say what exactly is missing about G. schweinfurthii. Similarly, the aim of study is too much broad. Rewrite the specified aims of study.

Materials and Methods:

 Under the heading of Isolation of the major compound, please explain choice of solvents for elution (CHCl3-MeOH).

 The name/code of the isolated compound (AL-03) is given, but no brief description of its identity is provided in Methods (could state structure elucidation will follow using NMR).

 Please clarify species and strain of mice used for toxicity testing.

 Please provide justification for using 2000 mg/kg as initial dose.

 Explain if ethical approval was obtained for animal experiments.

 Please Provide route of administration consistently for all groups.

 Mention randomization method used to allocate animals to groups.

 In Acetic Acid-Induced Writhing, please specify ethical considerations (minimizing animal suffering) and mention replication or whether multiple experiments were conducted.

 In Hot Plate method, please indicate sample size justification.

 For Carrageenan-Induced Paw Edema, please Include precise measurement technique for paw volume (plethysmometer vs. water displacement).

 Please M=mention if the experiment was performed blind to treatment groups.

 Please include statements regarding sample size (n), F-statistic with degrees of freedom, and exact p-values for ANOVA in the section of Statistical Analysis and apply this stat as well in the results.

Results and Discussion:

 LD50 was not determined exactly and claimed result is a preliminary acute toxicity assessment. This prevents misinterpretation as a formal LD50 study. Please find LD50 and give its detailed methodology as well in methods section.

 In section “TLC Analysis of 4-(2''-(4'-isopropylphenyl)propan-2''-yl)-2,3-dihydrofuran” please clarify whether the reported Rf values correspond to a purified compound or crude fraction.

 Please provide the rationale for selecting the two solvent systems (chloroform:methanol and chloroform:ethyl acetate).

 Consider reporting TLC plates’ reproducibility and whether co-TLC with related compounds was performed.

 Results in section “Analgesic activity of root extract using acetic acid-induced writhing method” It is unclear whether the 100 mg/kg dose showed any statistically significant effect compared to the negative control. This should be clarified.

 Please include in result paragraph with proper df and F-values along with P values.

 Please improve the discussion by clearly separating the results from explanations, avoiding strong assumptions about how the extract works, and making a clearer comparison with morphine and previous studies to better explain the central pain-relieving effects.

Conclusion:

 Rewrite the conclusion by keeping in mind that the results come from animal experiments, not human studies and also the isolated compound’s effects are not proven because it was not tested on its own.

 Include study limitations and recommend further experiments to confirm the findings.

References:

 Well documented

Concluding Remarks:

The manuscript has potential, but it can only be considered for publication after major revisions addressing the issues highlighted above.

**********

6. PLOS authors have the option to publish the peer review history of their article (what does this mean?). If published, this will include your full peer review and any attached files.

If you choose “no”, your identity will remain anonymous but your review may still be made public.

**Do you want your identity to be public for this peer review?**  For information about this choice, including consent withdrawal, please see our Privacy Policy.

Reviewer #1: **Yes:** Dr. Kanwal Ashiq

Reviewer #2: **Yes:** Dr. Aamir Mushtaq

**********

[NOTE: If reviewer comments were submitted as an attachment file, they will be attached to this email and accessible via the submission site. Please log into your account, locate the manuscript record, and check for the action link "View Attachments". If this link does not appear, there are no attachment files.]

To ensure your figures meet our technical requirements, please review our figure guidelines: https://journals.plos.org/plosone/s/figures

You may also use PLOS’s free figure tool, NAAS, to help you prepare publication quality figures: https://journals.plos.org/plosone/s/figures#loc-tools-for-figure-preparation.

NAAS will assess whether your figures meet our technical requirements by comparing each figure against our figure specifications.

# Author comment: Author response to Decision Letter 1

**DOI:** 10.1371/journal.pone.0353400.r002

18 Mar 2026

We thank the Academic Editor and reviewers for their valuable comments. All concerns have been addressed in the revised manuscript. A detailed point-by-point response is provided in the uploaded “Response to Reviewers” document.

1. Please amend your authorship list in your manuscript file to include author Abdi leta Gemechu.

− Response: Thank you for notifying us. The authorship list in the manuscript has now been updated to include Abdi Leta Gemechu as requested. The corrected authorship information has been incorporated into the revised manuscript.

2. Please ensure that you refer to Figure 2 in your text as, if accepted, production will need this reference to link the reader to the figure.

− Response: Thank you for bringing this to our attention. The manuscript has been revised to ensure that Figure 2 is properly cited in the main text, allowing correct linking between the text and the figure during production.

3. To comply with PLOS One submissions requirements, in your Methods section, please provide additional information regarding the experiments involving animals and ensure you have included details on (1) methods of sacrifice, (2) methods of anesthesia and/or analgesia, and (3) efforts to alleviate suffering.

4. To comply with PLOS One submissions requirements, in your Methods section, please provide additional information regarding the experiments involving animals and ensure you have included details on (1) methods of sacrifice, (2) methods of anesthesia and/or analgesia, and (3) efforts to alleviate suffering.

− Response: Thank you for the notification. The Methods section has been revised to include additional details regarding the animal experiments, including the methods of sacrifice, anesthesia/analgesia procedures, and measures taken to minimize pain and distress in accordance with PLOS ONE requirements and ethical guidelines.

5. We note that this submission includes NMR spectroscopy data. We would recommend that you include the following information in your methods section or as Supporting Information files:

a) The make/source of the NMR instrument used in your study, as well as the magnetic field strength. For each individual experiment, please also list: the nucleus being measured; the sample concentration; the solvent in which the sample is dissolved and if solvent signal suppression was used; the reference standard and the temperature.

b) A list of the chemical shifts for all compounds characterized by NMR spectroscopy, specifying, where relevant: the chemical shift (δ), the multiplicity and the coupling constants (in Hz), for the appropriate nuclei used for assignment.

c)The full integrated NMR spectrum, clearly labelled with the compound name and chemical structure.

− Response: Thank you for this helpful suggestion. The Methods section has been expanded to include detailed information regarding the NMR experiments. Specifically, we now report the instrument make and model, magnetic field strength, nuclei measured, sample concentration, solvent system, reference standard, temperature, and acquisition conditions.

− Response: In addition, the chemical shift data for the isolated compound are now presented following the standard reporting format, including δ values, signal multiplicities, and coupling constants (J, Hz). Furthermore, the complete ¹H NMR, ¹³C NMR, and HSQC spectra have been provided as Supporting Information, with each spectrum clearly labeled with the compound name and corresponding chemical structure.

7. We note that one or more of the authors is affiliated with the funding organization, indicating the funder may have had some role in the design, data collection, analysis or preparation of your manuscript for publication; in other words, the funder played an indirect role through the participation of the co-authors. If the funding organization did not play a role in the study design, data collection and analysis, decision to publish, or preparation of the manuscript and only provided financial support in the form of authors' salaries and/or research materials, please do the following:

1. Review your statements relating to the author contributions, and ensure you have specifically and accurately indicated the role(s) that these authors had in your study. These amendments should be made in the online form.

2. Confirm in your cover letter that you agree with the following statement, and we will change the online submission form on your behalf:

− Response: In accordance with the journal’s request, all funding-related text has been removed from the manuscript. The Funding Statement has been revised to clarify that the study was supported by the Office of the Vice President for Research and Innovation, Addis Ababa University (Grant No. TR/014/2021). Although one or more authors are affiliated with the funding institution, the funder did not have any additional role in the study design, data collection and analysis, decision to publish, or preparation of the manuscript beyond the contributions of the authors themselves. The specific roles of the authors are detailed in the Author Contributions section.

Attachment Submitted filename: Response_to_Reviewers_PONE-D-26-01189.docx

# Peer review: Decision Letter 1

**DOI:** 10.1371/journal.pone.0353400.r003

**Authors:** Eshetie Melese Birru

6 May 2026

PONE-D-26-01189R1

Evaluation of analgesic and anti-inflammatory activities of the root extract of Grewia schweinfurthii Burret and its major chemical constituents

PLOS One

Dear Dr. Gemechu,

Thank you for submitting your manuscript to PLOS ONE. After careful consideration, we feel that it has merit but does not fully meet PLOS ONE’s publication criteria as it currently stands. Therefore, we invite you to submit a revised version of the manuscript that addresses the points raised during the review process.

Please submit your revised manuscript by Jun 20 2026 11:59PM. If you will need more time than this to complete your revisions, please reply to this message or contact the journal office at plosone@plos.org. When you're ready to submit your revision, log on to https://www.editorialmanager.com/pone/ and select the 'Submissions Needing Revision' folder to locate your manuscript file.

Please include the following items when submitting your revised manuscript:

- A letter that responds to each point raised by the academic editor and reviewer(s). You should upload this letter as a separate file labeled 'Response to Reviewers'.
- A marked-up copy of your manuscript that highlights changes made to the original version. You should upload this as a separate file labeled 'Revised Manuscript with Track Changes'.
- An unmarked version of your revised paper without tracked changes. You should upload this as a separate file labeled 'Manuscript'.

If you would like to make changes to your financial disclosure, please include your updated statement in your cover letter. Guidelines for resubmitting your figure files are available below the reviewer comments at the end of this letter.

If applicable, we recommend that you deposit your laboratory protocols in protocols.io to enhance the reproducibility of your results. Protocols.io assigns your protocol its own identifier (DOI) so that it can be cited independently in the future. For instructions see: https://journals.plos.org/plosone/s/submission-guidelines#loc-laboratory-protocols. Additionally, PLOS ONE offers an option for publishing peer-reviewed Lab Protocol articles, which describe protocols hosted on protocols.io. Read more information on sharing protocols at https://plos.org/protocols?utm_medium=editorial-email&utm_source=authorletters&utm_campaign=protocols.

As the corresponding author, your ORCID iD is verified in the submission system and will appear in the published article. PLOS supports the use of ORCID, and we encourage all coauthors to register for an ORCID iD and use it as well. Please encourage your coauthors to verify their ORCID iD within the submission system before final acceptance, as unverified ORCID iDs will not appear in the published article. *Only*  the individual author can complete the verification step; PLOS staff *cannot*  verify ORCID iDs on behalf of authors.

We look forward to receiving your revised manuscript.

Kind regards,

Eshetie Melese Birru, PhD

Academic Editor

PLOS One

Journal Requirements:

If the reviewer comments include a recommendation to cite specific previously published works, please review and evaluate these publications to determine whether they are relevant and should be cited. There is no requirement to cite these works unless the editor has indicated otherwise.

Reviewers' comments:

Reviewer's Responses to Questions

**Comments to the Author**

1. If the authors have adequately addressed your comments raised in a previous round of review and you feel that this manuscript is now acceptable for publication, you may indicate that here to bypass the “Comments to the Author” section, enter your conflict of interest statement in the “Confidential to Editor” section, and submit your "Accept" recommendation.

Reviewer #1: All comments have been addressed

Reviewer #3: (No Response)

**********

2. Is the manuscript technically sound, and do the data support the conclusions?

The manuscript must describe a technically sound piece of scientific research with data that supports the conclusions. Experiments must have been conducted rigorously, with appropriate controls, replication, and sample sizes. The conclusions must be drawn appropriately based on the data presented.

Reviewer #1: Yes

Reviewer #3: Yes

**********

3. Has the statistical analysis been performed appropriately and rigorously?

Reviewer #1: Yes

Reviewer #3: Yes

**********

4. Have the authors made all data underlying the findings in their manuscript fully available?

The PLOS Data policy requires authors to make all data underlying the findings described in their manuscript fully available without restriction, with rare exception (please refer to the Data Availability Statement in the manuscript PDF file). The data should be provided as part of the manuscript or its supporting information, or deposited to a public repository. For example, in addition to summary statistics, the data points behind means, medians and variance measures should be available. If there are restrictions on publicly sharing data—e.g. participant privacy or use of data from a third party—those must be specified.

Reviewer #1: Yes

Reviewer #3: Yes

**********

5. Is the manuscript presented in an intelligible fashion and written in standard English?

PLOS ONE does not copyedit accepted manuscripts, so the language in submitted articles must be clear, correct, and unambiguous. Any typographical or grammatical errors should be corrected at revision, so please note any specific errors here.

Reviewer #1: Yes

Reviewer #3: Yes

**********

6. Review Comments to the Author

Please use the space provided to explain your answers to the questions above. You may also include additional comments for the author, including concerns about dual publication, research ethics, or publication ethics. (Please upload your review as an attachment if it exceeds 20,000 characters)

Reviewer #1: This manuscript presents a well-designed experimental pharmacological study with a strong methodological framework and meaningful biological outcomes. The investigation appropriately evaluates both the crude extract and isolated compound of Grewia schweinfurthii, which adds scientific depth and enhances the overall value of the work. The study demonstrates good use of validated in vivo models for analgesic and anti-inflammatory assessment, supporting the reliability of the findings.

Minor comments are given below:

The research gap and novelty justification could be more sharply defined at the end of the introduction to better position the study.

Justification for selection of isolated compound (AL-03) needs clarification in methods.

Limited integration between phytochemical constituents and observed biological activities, along with insufficient comparative discussion within the Results section.

The conclusion is appropriate and consistent with the study findings. It correctly highlights analgesic and anti-inflammatory potential of the extract and isolated compound. However, the unique contribution of dual evaluation (extract + compound) should be explicitly stated as the key highlight of the study.

Reviewer #3: (No Response)

**********

7. PLOS authors have the option to publish the peer review history of their article (what does this mean?). If published, this will include your full peer review and any attached files.

If you choose “no”, your identity will remain anonymous but your review may still be made public.

**Do you want your identity to be public for this peer review?**  For information about this choice, including consent withdrawal, please see our Privacy Policy.

Reviewer #1: **Yes:** Dr. Kanwal Ashiq

Reviewer #3: No

**********

[NOTE: If reviewer comments were submitted as an attachment file, they will be attached to this email and accessible via the submission site. Please log into your account, locate the manuscript record, and check for the action link "View Attachments". If this link does not appear, there are no attachment files.]

To ensure your figures meet our technical requirements, please review our figure guidelines: https://journals.plos.org/plosone/s/figures

You may also use PLOS’s free figure tool, NAAS, to help you prepare publication quality figures: https://journals.plos.org/plosone/s/figures#loc-tools-for-figure-preparation.

NAAS will assess whether your figures meet our technical requirements by comparing each figure against our figure specifications.

Attachment Submitted filename: Peer Review.docx

# Author comment: Author response to Decision Letter 2

**DOI:** 10.1371/journal.pone.0353400.r004

20 May 2026

REVIEWER #1

General Comment: This manuscript presents a well-designed experimental pharmacological study with a strong methodological framework and meaningful biological outcomes. The investigation appropriately evaluates both the crude extract and isolated compound of Grewia schweinfurthii, which adds scientific depth. The study demonstrates good use of validated in vivo models for analgesic and anti-inflammatory assessment.

Authors’ Response: We thank Reviewer #1 for the positive overall assessment and for the recognition of the study design and methodological rigor. We have carefully addressed each specific comment below.

Comment 1: The research gap and novelty justification could be more sharply defined at the end of the introduction to better position the study.

Authors’ Response: We have revised the final paragraph of the Introduction to more explicitly state the research gap and novelty. We have added text clarifying that no prior study has simultaneously evaluated both a crude extract and a purified bioactive compound from G. schweinfurthii using complementary peripheral, central, and inflammatory pain models. The revised passage reads:

"Although several species of the genus Grewia have been investigated pharmacologically, there is no prior experimental evidence validating the analgesic and anti-inflammatory activities of G. schweinfurthii root extract or its isolated constituents. Furthermore, no study to date has simultaneously evaluated both the crude extract and a purified bioactive compound from this species using validated peripheral, central, and inflammatory pain models. The present study therefore addresses this gap by providing the first systematic pharmacological characterization of G. schweinfurthii root and its major isolated compound, AL-03, using complementary in vivo models — representing the first dual extract-compound pharmacological evaluation of this species."

Comment 2: Justification for selection of isolated compound (AL-03) needs clarification in methods.

Authors’ Response: We have added a clarifying passage in the Isolation section of the Methods explaining that AL-03 was selected based on three criteria: (i) it was the principal compound isolated from the active chromatographic fractions; (ii) TLC analysis in two independent solvent systems confirmed chromatographic purity; and (iii) it was the highest-yielding isolate, suggesting it is a major constituent of the root extract. The added text reads:

"AL-03 was selected for further pharmacological evaluation based on three criteria: (i) it was the principal compound isolated from the active chromatographic fractions (fractions 21–30); (ii) TLC analysis in two independent solvent systems (CHCl₃:MeOH 6:1 and CHCl₃:EtOAc 2:1) consistently yielded a single spot, confirming chromatographic purity; and (iii) it represented the highest-yielding isolate from column chromatography, suggesting it is a major constituent of the root extract."

Comment 3: Limited integration between phytochemical constituents and observed biological activities, along with insufficient comparative discussion within the Results section.

Authors’ Response: We have expanded the Discussion to more explicitly link detected phytochemical classes to observed activities. Flavonoids and terpenoids are linked to COX/LOX pathway inhibition consistent with late-phase carrageenan data; tannins and saponins are linked to peripheral analgesic activity via suppression of inflammatory mediators. We have also expanded comparative discussion between extract efficacy and reference drugs, and contextualized the AL-03 findings relative to the extract data. These additions are incorporated in the Cohesive Integration subsection and within individual results subsections.

Comment 4: The conclusion is appropriate and consistent with the study findings. However, the unique contribution of dual evaluation (extract + compound) should be explicitly stated as the key highlight of the study.

Authors’ Response: The Conclusion has been revised to explicitly highlight the dual-evaluation approach. The following sentence has been added:

"Importantly, the simultaneous evaluation of both a crude extract and a structurally characterized isolated compound within a single study provides a scientifically rigorous framework for linking observed pharmacological effects to specific chemical entities. This dual extract–compound approach represents a key methodological strength of this work and constitutes a meaningful contribution to the growing literature on African medicinal plants."

Attachment Submitted filename: Response_to_Reviewers.docx

# Peer review: Decision Letter 2

**DOI:** 10.1371/journal.pone.0353400.r005

**Authors:** Eshetie Melese Birru

4 Jun 2026

PONE-D-26-01189R2

Evaluation of analgesic and anti-inflammatory activities of the root extract of Grewia schweinfurthii Burret and its major chemical constituents

PLOS One

Dear Dr. Gemechu,

Thank you for submitting your manuscript to PLOS ONE. After careful consideration, we feel that it has merit but does not fully meet PLOS ONE’s publication criteria as it currently stands. Therefore, we invite you to submit a revised version of the manuscript that addresses the points raised during the review process.

Please submit your revised manuscript by Jul 19 2026 11:59PM. If you will need more time than this to complete your revisions, please reply to this message or contact the journal office at plosone@plos.org. When you're ready to submit your revision, log on to https://www.editorialmanager.com/pone/ and select the 'Submissions Needing Revision' folder to locate your manuscript file.

Please include the following items when submitting your revised manuscript:

- A letter that responds to each point raised by the academic editor and reviewer(s). You should upload this letter as a separate file labeled 'Response to Reviewers'.
- A marked-up copy of your manuscript that highlights changes made to the original version. You should upload this as a separate file labeled 'Revised Manuscript with Track Changes'.
- An unmarked version of your revised paper without tracked changes. You should upload this as a separate file labeled 'Manuscript'.

If you would like to make changes to your financial disclosure, please include your updated statement in your cover letter. Guidelines for resubmitting your figure files are available below the reviewer comments at the end of this letter.

If applicable, we recommend that you deposit your laboratory protocols in protocols.io to enhance the reproducibility of your results. Protocols.io assigns your protocol its own identifier (DOI) so that it can be cited independently in the future. For instructions see: https://journals.plos.org/plosone/s/submission-guidelines#loc-laboratory-protocols. Additionally, PLOS ONE offers an option for publishing peer-reviewed Lab Protocol articles, which describe protocols hosted on protocols.io. Read more information on sharing protocols at https://plos.org/protocols?utm_medium=editorial-email&utm_source=authorletters&utm_campaign=protocols.

As the corresponding author, your ORCID iD is verified in the submission system and will appear in the published article. PLOS supports the use of ORCID, and we encourage all coauthors to register for an ORCID iD and use it as well. Please encourage your coauthors to verify their ORCID iD within the submission system before final acceptance, as unverified ORCID iDs will not appear in the published article. *Only*  the individual author can complete the verification step; PLOS staff *cannot*  verify ORCID iDs on behalf of authors.

We look forward to receiving your revised manuscript.

Kind regards,

Eshetie Melese Birru, PhD

Academic Editor

PLOS One

**Journal Requirements:**

If the reviewer comments include a recommendation to cite specific previously published works, please review and evaluate these publications to determine whether they are relevant and should be cited. There is no requirement to cite these works unless the editor has indicated otherwise.

[Note: HTML markup is below. Please do not edit.]

Reviewers' comments:

Reviewer's Responses to Questions

**Comments to the Author**

1. If the authors have adequately addressed your comments raised in a previous round of review and you feel that this manuscript is now acceptable for publication, you may indicate that here to bypass the “Comments to the Author” section, enter your conflict of interest statement in the “Confidential to Editor” section, and submit your "Accept" recommendation.

Reviewer #1: All comments have been addressed

Reviewer #3: (No Response)

**********

2. Is the manuscript technically sound, and do the data support the conclusions?

The manuscript must describe a technically sound piece of scientific research with data that supports the conclusions. Experiments must have been conducted rigorously, with appropriate controls, replication, and sample sizes. The conclusions must be drawn appropriately based on the data presented.

Reviewer #1: Yes

Reviewer #3: Yes

**********

3. Has the statistical analysis been performed appropriately and rigorously?

Reviewer #1: Yes

Reviewer #3: Yes

**********

4. Have the authors made all data underlying the findings in their manuscript fully available?

The PLOS Data policy requires authors to make all data underlying the findings described in their manuscript fully available without restriction, with rare exception (please refer to the Data Availability Statement in the manuscript PDF file). The data should be provided as part of the manuscript or its supporting information, or deposited to a public repository. For example, in addition to summary statistics, the data points behind means, medians and variance measures should be available. If there are restrictions on publicly sharing data—e.g. participant privacy or use of data from a third party—those must be specified.

Reviewer #1: Yes

Reviewer #3: Yes

**********

5. Is the manuscript presented in an intelligible fashion and written in standard English?

PLOS ONE does not copyedit accepted manuscripts, so the language in submitted articles must be clear, correct, and unambiguous. Any typographical or grammatical errors should be corrected at revision, so please note any specific errors here.

Reviewer #1: Yes

Reviewer #3: Yes

**********

6. Review Comments to the Author

Please use the space provided to explain your answers to the questions above. You may also include additional comments for the author, including concerns about dual publication, research ethics, or publication ethics. (Please upload your review as an attachment if it exceeds 20,000 characters)

Reviewer #1: (No Response)

Reviewer #3: (No Response)

**********

7. PLOS authors have the option to publish the peer review history of their article (what does this mean?). If published, this will include your full peer review and any attached files.

If you choose “no”, your identity will remain anonymous but your review may still be made public.

**Do you want your identity to be public for this peer review?**  For information about this choice, including consent withdrawal, please see our Privacy Policy.

Reviewer #1: **Yes:** Dr. Kanwal Ashiq

Reviewer #3: **Yes:** Wêndeo Kennedy Costa

**********

[NOTE: If reviewer comments were submitted as an attachment file, they will be attached to this email and accessible via the submission site. Please log into your account, locate the manuscript record, and check for the action link "View Attachments". If this link does not appear, there are no attachment files.]

To ensure your figures meet our technical requirements, please review our figure guidelines: https://journals.plos.org/plosone/s/figures

You may also use PLOS’s free figure tool, NAAS, to help you prepare publication quality figures: https://journals.plos.org/plosone/s/figures#loc-tools-for-figure-preparation.

NAAS will assess whether your figures meet our technical requirements by comparing each figure against our figure specifications.

Attachment Submitted filename: Peer_Review_declet_2.docx

# Author comment: Author response to Decision Letter 3

**DOI:** 10.1371/journal.pone.0353400.r006

8 Jun 2026

Response to Reviewer Comments:

We thank the Academic Editor and Peer Reviewer for their comprehensive critique. All 21 comments have been addressed below. All changes are highlighted in the tracked-changes manuscript.

Comment 1: Introduction revised to clarify that no prior study has evaluated G. schweinfurthii root extract specifically, that AL-03 is newly reported from this species, and that the simultaneous dual extract–compound evaluation represents a methodological advance.

Comment 2: Methods and Discussion revised to clarify AL-03 selection rationale. The Discussion now explicitly states that AL-03 cannot be confirmed as the sole contributor to extract activity and that synergistic phytochemical interactions likely contribute.

Comment 3: Acknowledged as a limitation. Quantitative profiling of total phenolics and flavonoids was not performed and is identified as a priority for future work in the new Limitations section.

Comment 4: Acknowledged as a limitation. Structural assignment relied on ¹H NMR, ¹³C NMR, and HSQC data; absence of HRMS or LC-MS/MS confirmation is stated in the Limitations section.

Comment 5: Discussion revised to clarify that the 40-fold dose difference between extract (400 mg/kg) and indomethacin (10 mg/kg), combined with the absence of standardization, precludes any potency equivalence claim.

Comment 6: Methods now states that formal a priori power analysis was not performed and that sample size was based on established practice in comparable studies. Noted in the Limitations section.

Comment 7: Methods now explicitly state that animals were allocated by random number assignment and all behavioral assessments were conducted by a blinded investigator.

Comment 8: Acknowledged. Sex was distributed evenly across groups, but no formal sex-stratified analysis was performed. Stated in both the Methods and Limitations sections.

Comment 9: Dose selection rationale (fractions of the 2000 mg/kg limit dose) is now clearly explained. Absence of a full dose-response curve and ED50 is acknowledged as a limitation.

Comment 10: All mechanistic statements revised to include explicit qualifiers ("consistent with," "may plausibly involve," "suggests but does not confirm"). Limitations section confirms no direct biochemical assays were performed.

Comment 11: Discussion now contextualizes the 93% inhibition relative to the 40-fold dose difference, notes the model's validated sensitivity, and frames findings as preliminary proof-of-concept.

Comment 12: Acknowledged as a statistical limitation. One-way ANOVA applied at individual time points does not account for within-subject correlation; repeated-measures ANOVA would be more appropriate. Noted in the Statistical Analysis and Limitations sections.

Comment 13: Statistical Analysis section now distinguishes p-values from effect magnitude. Absence of reported effect sizes acknowledged as a limitation.

Comment 14: 95% confidence intervals have been calculated and added to all relevant tables in the revised manuscript.

Comment 15: All mechanistic conclusions revised throughout. Language implying confirmed COX inhibition replaced with appropriately hedged inferences requiring biochemical confirmation.

Comment 16: New paragraph added to the Discussion interpreting the biphasic response. Higher inhibition in the late phase (3–5 h; 84–93% at 400 mg/kg) versus early phase (71%) suggests the extract's activity aligns more closely with prostaglandin-mediated pathways.

Comment 17: All references to therapeutic applicability revised. The Conclusion now states that findings are animal-based and preliminary, and that pharmacokinetic, bioavailability, and sub-chronic toxicity studies are prerequisites before any translational inference.

Comment 18: Statement corrected to: "While acute inflammation is a necessary and beneficial component of host defense, a dysregulated or excessively prolonged response can lead to collateral tissue injury and contribute to worse disease outcomes."

Comment 19: Environmental conditions, extraction yield (12.6% w/w), drying conditions, and storage details added to the relevant Methods sections to ensure reproducibility.

Comment 20: Discussion substantially revised to include comparative contextualization with other Grewia species, more critical discussion of effect sizes, explicit phytochemical–pharmacological integration, and a new Cohesive Integration subsection.

Comment 21: A dedicated Limitations section has been added enumerating nine key limitations: (1) acute models only; (2) qualitative phytochemical characterization; (3) no HRMS confirmation for AL-03; (4) no pharmacokinetic/toxicokinetic data; (5) no direct mechanistic assays; (6) sample size by convention not power analysis; (7) no sex stratification; (8) one-way ANOVA on time-course data; and (9) no extract standardization.

Attachment Submitted filename: Response_to_Reviewers_auresp_3.docx

# Peer review: Decision Letter 3

**DOI:** 10.1371/journal.pone.0353400.r007

**Authors:** Eshetie Melese Birru

23 Jun 2026

Evaluation of analgesic and anti-inflammatory activities of the root extract of Grewia schweinfurthii Burret and its major chemical constituents

PONE-D-26-01189R3

Dear Dr. Gemechu,

We’re pleased to inform you that your manuscript has been judged scientifically suitable for publication and will be formally accepted for publication once it meets all outstanding technical requirements.

Within one week, you’ll receive an e-mail detailing the required amendments. When these have been addressed, you’ll receive a formal acceptance letter and your manuscript will be scheduled for publication.

An invoice will be generated when your article is formally accepted. Please note, if your institution has a publishing partnership with PLOS and your article meets the relevant criteria, all or part of your publication costs will be covered. Please make sure your user information is up-to-date by logging into Editorial Manager at Editorial Manager® and clicking the ‘Update My Information' link at the top of the page. For questions related to billing, please contact billing support.

If your institution or institutions have a press office, please notify them about your upcoming paper to help maximize its impact. If they’ll be preparing press materials, please inform our press team as soon as possible -- no later than 48 hours after receiving the formal acceptance. Your manuscript will remain under strict press embargo until 2 pm Eastern Time on the date of publication. For more information, please contact onepress@plos.org.

Kind regards,

Eshetie Melese Birru, PhD

Academic Editor

PLOS One

Reviewers' comments:

Reviewer's Responses to Questions

**Comments to the Author**

1. If the authors have adequately addressed your comments raised in a previous round of review and you feel that this manuscript is now acceptable for publication, you may indicate that here to bypass the “Comments to the Author” section, enter your conflict of interest statement in the “Confidential to Editor” section, and submit your "Accept" recommendation.

Reviewer #3: All comments have been addressed

**********

2. Is the manuscript technically sound, and do the data support the conclusions?

The manuscript must describe a technically sound piece of scientific research with data that supports the conclusions. Experiments must have been conducted rigorously, with appropriate controls, replication, and sample sizes. The conclusions must be drawn appropriately based on the data presented.

Reviewer #3: Yes

**********

3. Has the statistical analysis been performed appropriately and rigorously?

Reviewer #3: Yes

**********

4. Have the authors made all data underlying the findings in their manuscript fully available?

The PLOS Data policy requires authors to make all data underlying the findings described in their manuscript fully available without restriction, with rare exception (please refer to the Data Availability Statement in the manuscript PDF file). The data should be provided as part of the manuscript or its supporting information, or deposited to a public repository. For example, in addition to summary statistics, the data points behind means, medians and variance measures should be available. If there are restrictions on publicly sharing data—e.g. participant privacy or use of data from a third party—those must be specified.

Reviewer #3: Yes

**********

5. Is the manuscript presented in an intelligible fashion and written in standard English?

PLOS ONE does not copyedit accepted manuscripts, so the language in submitted articles must be clear, correct, and unambiguous. Any typographical or grammatical errors should be corrected at revision, so please note any specific errors here.

Reviewer #3: Yes

**********

6. Review Comments to the Author

Please use the space provided to explain your answers to the questions above. You may also include additional comments for the author, including concerns about dual publication, research ethics, or publication ethics. (Please upload your review as an attachment if it exceeds 20,000 characters)

Reviewer #3: (No Response)

**********

7. PLOS authors have the option to publish the peer review history of their article (what does this mean?). If published, this will include your full peer review and any attached files.

If you choose “no”, your identity will remain anonymous but your review may still be made public.

**Do you want your identity to be public for this peer review?**  For information about this choice, including consent withdrawal, please see our Privacy Policy.

Reviewer #3: **Yes:** Wêndeo Kennedy Costa

**********

# Editor report: Acceptance letter

**DOI:** 10.1371/journal.pone.0353400.r008

**Authors:** Eshetie Melese Birru

PONE-D-26-01189R3

PLOS One

Dear Dr. Gemechu,

I'm pleased to inform you that your manuscript has been deemed suitable for publication in PLOS One. Congratulations! Your manuscript is now being handed over to our production team.

At this stage, our production department will prepare your paper for publication. This includes ensuring the following:

* All references, tables, and figures are properly cited

* All relevant supporting information is included in the manuscript submission,

* There are no issues that prevent the paper from being properly typeset

You will receive further instructions from the production team, including instructions on how to review your proof when it is ready. Please keep in mind that we are working through a large volume of accepted articles, so please give us a few days to review your paper and let you know the next and final steps.

Lastly, if your institution or institutions have a press office, please let them know about your upcoming paper now to help maximize its impact. If they'll be preparing press materials, please inform our press team within the next 48 hours. Your manuscript will remain under strict press embargo until 2 pm Eastern Time on the date of publication. For more information, please contact onepress@plos.org.

You will receive an invoice from PLOS for your publication fee after your manuscript has reached the completed accept phase. If you receive an email requesting payment before acceptance or for any other service, this may be a phishing scheme. Learn how to identify phishing emails and protect your accounts at https://explore.plos.org/phishing.

If we can help with anything else, please email us at customercare@plos.org.

Thank you for submitting your work to PLOS ONE and supporting open access.

Kind regards,

PLOS ONE Editorial Office Staff

on behalf of

Dr. Eshetie Melese Birru

Academic Editor

PLOS One
